# Immunoprofiling of active and inactive systemic juvenile idiopathic arthritis reveals distinct biomarkers: a single-center study

**DOI:** 10.1186/s12969-021-00660-9

**Published:** 2021-12-28

**Authors:** Heshuang Qu, Erik Sundberg, Cecilia Aulin, Manoj Neog, Karin Palmblad, Anna Carin Horne, Fredrik Granath, Alexandra Ek, Erik Melén, Mia Olsson, Helena Erlandsson Harris

**Affiliations:** 1 Department of Medicine Solna, Center for Molecular Medicine, Karolinska Institutet, Stockholm, Sweden; 2 Division of Rheumatology, Karolinska University Hospital, Stockholm, Sweden; 3 Unit of Pediatric Rheumatology, Karolinska University Hospital, Stockholm, Sweden; 4 Department of Women’s and Children’s Health, Karolinska Institutet, Stockholm, Sweden; 5 Division of Clinical Epidemiology, Department of Medicine Solna, Karolinska Institutet, Stockholm, Sweden; 6 Center for Occupational and Environmental Medicine, Region Stockholm, Stockholm, Sweden; 7 Sachs Children’s Hospital, Stockholm, Sweden; 8 Department of Clinical Sciences and Education, Karolinska Institutet, Södersjukhuset, Stockholm, Sweden

**Keywords:** Systemic juvenile idiopathic arthritis, Cytokines and inflammatory mediators, Inflammation, Proteomics, Ingenuity pathway analysis, High mobility group Box 1

## Abstract

**Background:**

This study aimed to perform an immunoprofiling of systemic juvenile idiopathic arthritis (sJIA) in order to define biomarkers of clinical use as well as reveal new immune mechanisms.

**Methods:**

Immunoprofiling of plasma samples from a clinically well-described cohort consisting of 21 sJIA patients as well as 60 age and sex matched healthy controls, was performed by a highly sensitive proteomic immunoassay. Based on the biomarkers being significantly up- or down-regulated in cross-sectional and paired analysis, related canonical pathways and cellular functions were explored by Ingenuity Pathway Analysis (IPA).

**Results:**

The well-studied sJIA biomarkers, IL6, IL18 and S100A12, were confirmed to be increased during active sJIA as compared to healthy controls. IL18 was the only factor found to be increased during inactive sJIA as compared to healthy controls. Novel factors, including CASP8, CCL23, CD6, CXCL1, CXCL11, CXCL5, EIF4EBP1, KITLG, MMP1, OSM, SIRT2, SULT1A1 and TNFSF11, were found to be differentially expressed in active and/or inactive sJIA and healthy controls. No significant pathway activation could be predicted based on the limited factor input to the IPA. High Mobility Group Box 1 (HMGB1), a damage associated molecular pattern being involved in a series of inflammatory diseases, was determined to be higher in active sJIA than inactive sJIA.

**Conclusions:**

We could identify a novel set of biomarkers distinguishing active sJIA from inactive sJIA or healthy controls. Our findings enable a better understanding of the immune mechanisms active in sJIA and aid the development of future diagnostic and therapeutic strategies.

**Supplementary Information:**

The online version contains supplementary material available at 10.1186/s12969-021-00660-9.

## Background

Systemic juvenile idiopathic arthritis (sJIA) accounts for 4–17% of juvenile idiopathic arthritis (JIA) cases. It is distinguished from the other JIA subtypes by its clinical features, pathogenetic mechanisms and by treatment protocols used [1]. Hallmarks of sJIA are chronic arthritis accompanied by high spiking fever that lasts for at least two weeks. Additional systemic symptoms may include rheumatic rash, hepatosplenomegaly, lymphadenopathy and serositis [2]. There is no pathognomonic feature that distinguishes sJIA from other conditions but few laboratory parameters may be supportive for diagnosis of sJIA, such as elevated C-reactive protein (CRP), erythrocyte sedimentation rate (ESR), neutrophil and platelet counts [3]. Manifestations of sJIA, including macrophage activation syndrome (MAS), neurological complications, limitation in functional outcome by arthritis and long-term damage from chronic inflammation can be severe and have significant impacts on patients’ quality of life [1]. The sJIA pathogenesis has been associated with dysregulation of the innate immune system, placing sJIA in the spectrum of autoinflammatory diseases rather than of autoimmune diseases [4].

Certain serological biomarkers have been linked to different features of sJIA. Interleukin (IL) 1 and IL6 is proposed to be critical in the pathogenesis of the disease and are also effectively targeted by treatments used for sJIA [5, 6]. High levels of serum IL6 measured in patients have been correlated with CRP, iron, hemoglobin, and platelet levels before treatment [7]. IL18 has been suggested to predict disease activity, to estimate disease severity and development of MAS [8]. The phagocyte-derived proteins S100A8/A9 and S100A12 proteins can be used as diagnostic biomarkers and as indicators of therapeutic responses [9, 10]. Soluble CD163 and IL2 are suggested to detect subclinical MAS and to predict the development of overt MAS in sJIA [11]. Lastly, Matrix Metalloproteinase 3 (MMP3) have been reported to correlate with the progression of structural joint damage in sJIA [12].

Currently, there is no cure for the disease and better clinical tools for diagnostics and prognostics are needed [1]. In this hypothesis-generating study, we determined the plasma levels of ninety-two proteins linked to inflammation in twenty-one sJIA patients with both active and inactive disease, as well as in age and sex matched healthy controls. Our aim was to identify biomarkers that could aid and improve disease diagnosis and to reveal underlying disease mechanisms and how they may contribute to sJIA flares and remission.

## Methods

### Patients

Plasma samples from twenty-one sJIA patients were collected at Astrid Lindgren’s Children Hospital in Stockholm, Sweden. Pediatric rheumatologists diagnosed all patients according to the International League of Associations for Rheumatology (ILAR) criteria [2]. The sJIA patients were 10 males and 11 females, with sampling ages ranging from 3 to 16 years. Disease activity was classified using the criteria approved by the American College of Rheumatology Board of Directors as Provisional [13]. None of the patients has MAS at the time when the samples were collected. Patients were considered as having inactive sJIA when they had no clinical signs of disease activity such as joints with active arthritis, fever, rash, serositis and splenomegaly or generalized lymphadenopathy attributable to sJIA. In addition, when available, ESR and CRP levels had to be within normal ranges and the physician’s global assessment of disease activity score had to be 0. Comprehensive clinical and laboratory data were collected at each plasma-sampling occasion.

Plasma samples from 60 healthy controls (four, eight or twelve years old) without chronic inflammatory or autoimmune diseases, were from a population-based cohort (Barnens miljö- och hälsoundersökning) in the Stockholm region [14].

Fresh blood from all subjects was collected in EDTA tubes, kept at room temperature and centrifuged within 4 h for 10–15 min at 3000–6000 g with brake. Centrifuged plasma was finally stored at − 80 °C until analysis.

### Proximity extension assay

Plasma samples were analyzed using a high-throughput, multiplex immunoassay (Proseek Multiplex, Proximity Extension Assay (PEA) technology, Inflammation panel, Olink Bioscience, Sweden). The biomarkers included in the inflammation panel are reported to be involved in multiple inflammatory conditions. The panel includes 92 immune-related proteins, primarily cytokines and chemokines as listed in Supplementary Table S1. The assay utilizes epitopespecific binding and hybridization of a set of paired oligonucleotide antibody probes, which is subsequently amplified using quantitative PCR, resulting in log base-2 normalized protein expression (NPX) values. Proteins with a call rate below 20% were excluded from further analysis, resulting in 69 proteins being included. Details regarding the design of the Inflammation panel, the PEA assay protocol and data pre-processing are outlined in supplementary files.

### HMGB1 quantification

Quantification of plasma HMGB1 was performed by Enzyme-Linked ImmunoSorbent Assay (ELISA) (IBL International, Germany) according to the manufacturer’s instructions.

### Data processing and statistical analysis

In the cross-sectional analysis, ordinary two-way ANOVA was performed on active sJIA (*n* = 14), inactive sJIA (*n* = 16) and healthy controls (*n* = 30). Multiple t-test was performed on active sJIA (n = 14) versus healthy controls (*n* = 28) and on inactive sJIA (n = 16) versus healthy controls (*n* = 32), separately. In each cross-sectional analysis, the healthy control group was age and sex matched to the patient group. In the paired analysis, two-way repeat-measurement ANOVA was performed on paired active sJIA (*n* = 9) and inactive sJIA (n = 9) samples from 9 patients. All the statistical analyses were corrected for multiple comparison by controlling the False Discovery Rate (FDR) via two-stage step-up method of Benjamini, Krieger and Yekutieli. Adjusted *p*-values less than 0.05 were regarded as significant. The tests were performed by using GraphPad Prism version 8.4.3 (San Diego, CA, USA).

Hierarchical Clustering Analysis (HCA) and Principal Component Analysis (PCA) analysis was performed by ClustVis Web Tool [15] to visualize the clustering of samples. Feature ranking process using Random Forest (RF) algorithm was executed using Python Jupyterlab 1.2.6.

Proteins with significant separation between two compared groups were analyzed for the enrichment in biological signaling pathways using Ingenuity Pathway Analysis (IPA) software (QIAGEN Inc.). Fold-change was calculated by dividing the average NPX value of sJIA (or active sJIA) by average NPX value of healthy controls (or inactive sJIA). Expression core analysis was based on fold-change of the statistically significantly separated proteins, including direct and indirect relationships, interaction and causal networks, all node types and data sources, experimentally observed confidence, restricted to human tissues and primary cells. Z-scores represent the predicted activation state of upstream regulators using the expression patterns of the downstream factors, based on relationships published in the literature. Statistical analysis was performed using Fisher’s exact test right-tailed within the IPA software.

Additional details regarding study design and statistical analysis are outlined in Supplementary Fig. S3 and Supplementary methods.

## Results

A summary of the demographics and disease characteristics of the study subjects are outlined in Table 1.

**Table 1:** Clinical characteristics of sJIA patients at the sampling time points

Patient #	Sex	Disease duration at sampling (months)	Sampling Age (years)	Symptoms at sampling	CRP (mg/dL)	Treatments at sampling	Treatments duration at sampling (months)	Analysis groups		
								Active	Inactive	Paired
**1**	M	15	3	arthritis	16	Prednisolone, MTX, Adalimumab	8, 15, 15	Yes		Yes
		24	4	NS	1	Tocilizumab, MTX	9, 24		Yes	
**2**	F	1	3	arthritis	87	Ibuprofen	1	Yes		Yes
		14	4	NS	< 1	NT			Yes	
**3**	M	4	4	fever, arthritis	193	Prednisolone	4	Yes		Yes
		81	7	NS	< 1	Canakinumab	26		Yes	
**4**	M	21	5	arthritis, fever, hepatomegaly, splenomegaly	123	MTX, Prednisolone, Canakinumab	1, 1, 7	Yes		Yes
		27	6	NS	NA	Canakinumab	13		Yes	
**5**	M	1	9	fever, splenomegaly, enthesopathy	52	Prednisolone	1	Yes		Yes
		4	10	NS	1	Canakinumab, Prednisolone	3, 4		Yes	
**6**	M	76	7	arthritis	< 1	NT		Yes		Yes
		105	10	NS	< 1	MTX, Tocilizumab	36, 44		Yes	
**7**	M	57	13	NS^*^	35	Tocilizumab	39	Yes		Yes
		72	14	NS	1	Tocilizumab	54		Yes	
**8**	F	63	14	fever, arthritis	33	Etanercept	12	Yes		Yes
		66	14	NS	9	Prednisolone, Canakinumab	2, 2		Yes	
**9**	F	32	7	rash, arthritis	< 1	Prednisolone, Tocilizumab	1, 24	Yes		Yes
		113	13	NS	1	NT			Yes	
**10**	F	3	10	fever, rash	1	Prednisolone	1	Yes		
**11**	M	1	10	fever, rash	21	NT		Yes		
**12**	M	30	14	arthritis	34	Anakinra	25	Yes		
**13**	F	23	16	fever	71	NT		Yes		
**14**	F	7	16	arthritis	< 1	Tocilizumab, MTX	3, 6	Yes		
**15**	F	47	6	NS	< 1	Canakinumab, MTX	3, 47		Yes	
**16**	M	5	8	NS	1	Prednisolone	4		Yes	
**17**	M	2	8	NS	NA	Prednisolone	1		Yes	
**18**	F	85	11	NS	1	NT			Yes	
**19**	F	95	13	NS	< 1	Anakinra, Prednisolone	5, 43		Yes	
**20**	F	2	13	NS	1	Prednisolone	1		Yes	
**21**	F	9	13	NS	NA	Prednisolone	8		Yes	

*The patient had no JIA-related symptom, but a CRP level of 35 mg/dL and generalized pain. According to the rheumatologists’ experience of this particular patients’ clinical history, the patient was regarded to be in an active disease phase

Abbreviations: CRP, C-reactive protein; MTX, Methotrexate; NA, data of CRP level was unavailable; NS, no clinical symptom; NT, no treatment ongoing

### Inflammation-associated proteins separate active sJIA, inactive sJIA and healthy controls

**Fig. 1:**
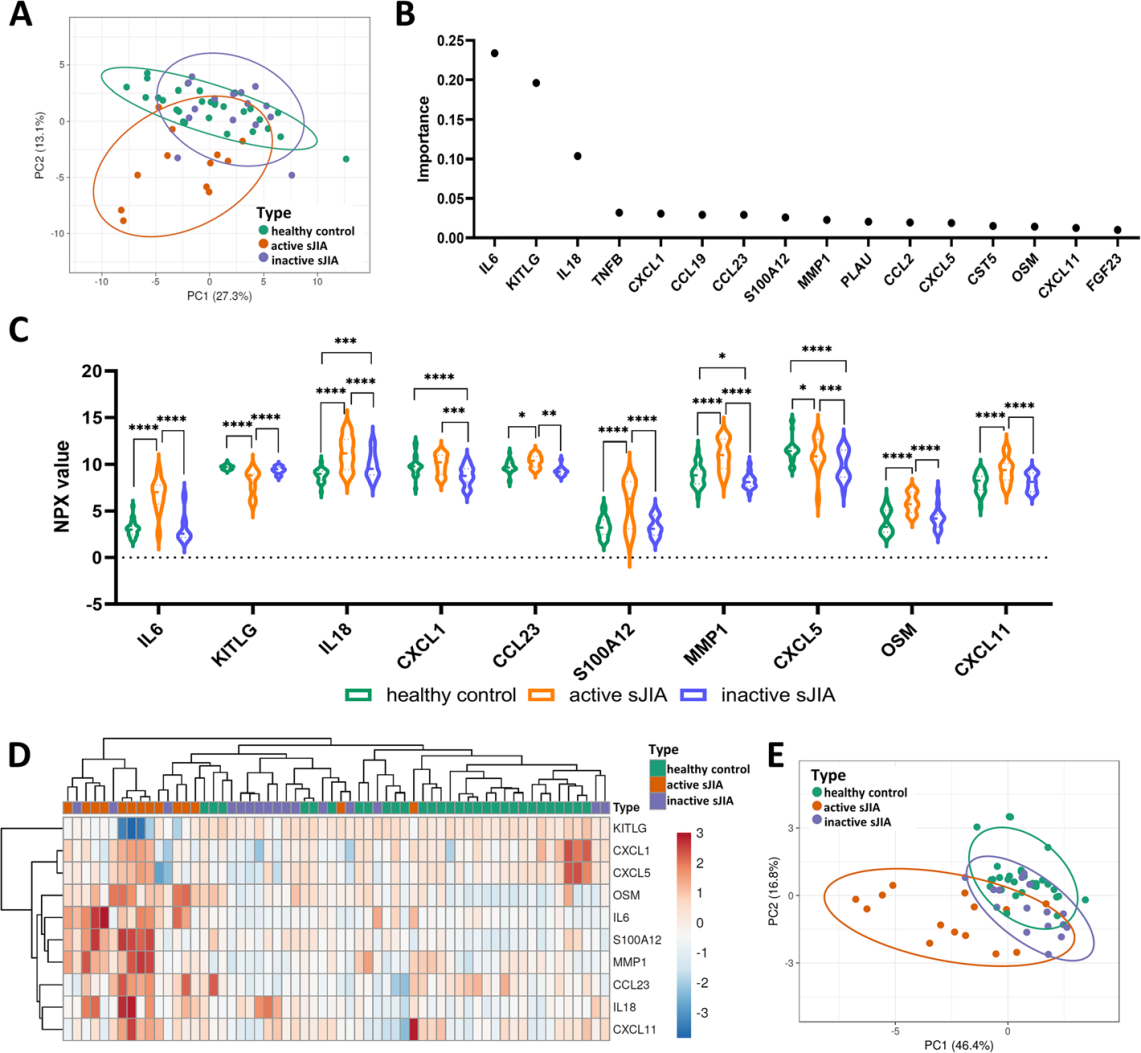
Distribution of the different subgroups based on detected inflammation-associated proteins**. A** Principal component analysis (PCA) of the three groups based on all included 69 proteins. Confidence level of the ellipses is 0.90. **B** Random forest analysis resulted in a predictive accuracy of 90.6%. The factor importance plot displays the top contributing proteins with importance higher than 0.01. **C** Comparative analysis of the proteins with top importance in (**B**) revealed significant difference among at least one of the three comparisons. Bars represent mean ± standard deviation. **D** Hierarchical clustering analysis based on the 10 proteins in (**C**) showing the grouping among active sJIA, inactive sJIA and controls. Unit variance scaling was applied to rows; both rows and columns were clustered using correlation distance and average linkage. **E** PCA of the three groups based on the 10 proteins shown in (**C**). The confidence level of the ellipses is 0.90. Statistics: ordinary two-way ANOVA with correction of multiple comparison by controlling the False Discovery Rate (FDR) of 5% via two-stage step-up method of Benjamini, Krieger and Yekutieli, * *p* < 0.05, ** *p* < 0.01, *** *p* < 0.001

Based on the 69 proteins included in the analysis, the groups of active sJIA, inactive sJIA and healthy controls could be identified but not completely separated by HCA (Supplementary Fig. S4). PCA also showed an overlap, with the active sJIA group more separated by principal components 1 and 2 (PC1 and PC2) from the inactive sJIA group and the healthy control group (Fig. 1A). Random forest analysis identified the proteins that contributed most strongly to the separation of the three groups (Fig. 1B, full data set of the cross-sectional analysis are outlined in Supplementary Table S3), with the top contributing proteins being IL6, KITLG, IL18, TNFB, CXCL1, CCL19, CCL23, S100A12, MMP1, PLAU, CCL2, CXCL5, CST5, OSM, CXCL11 and FGF23, each with importance larger than 0.01. Comparative analysis of the top contributing proteins demonstrated that, 10 of the 16 top proteins were significantly differentially expressed in at least one of the comparisons between the three groups (Fig. 1C). Active sJIA, inactive sJIA and healthy controls were further separated in HCA based on these 10 proteins than by using all 69 recorded proteins (Fig. 1D and Supplementary Fig. S4). Similarly, PCA based on the 10 differentially expressed proteins also resulted in better separation; in particular, the active sJIA group could be separated from inactive sJIA and healthy controls (Fig. 1E and A).

### Cross-sectional analysis identified proteins separating inactive sJIA or active sJIA group from healthy controls

**Fig. 2:**
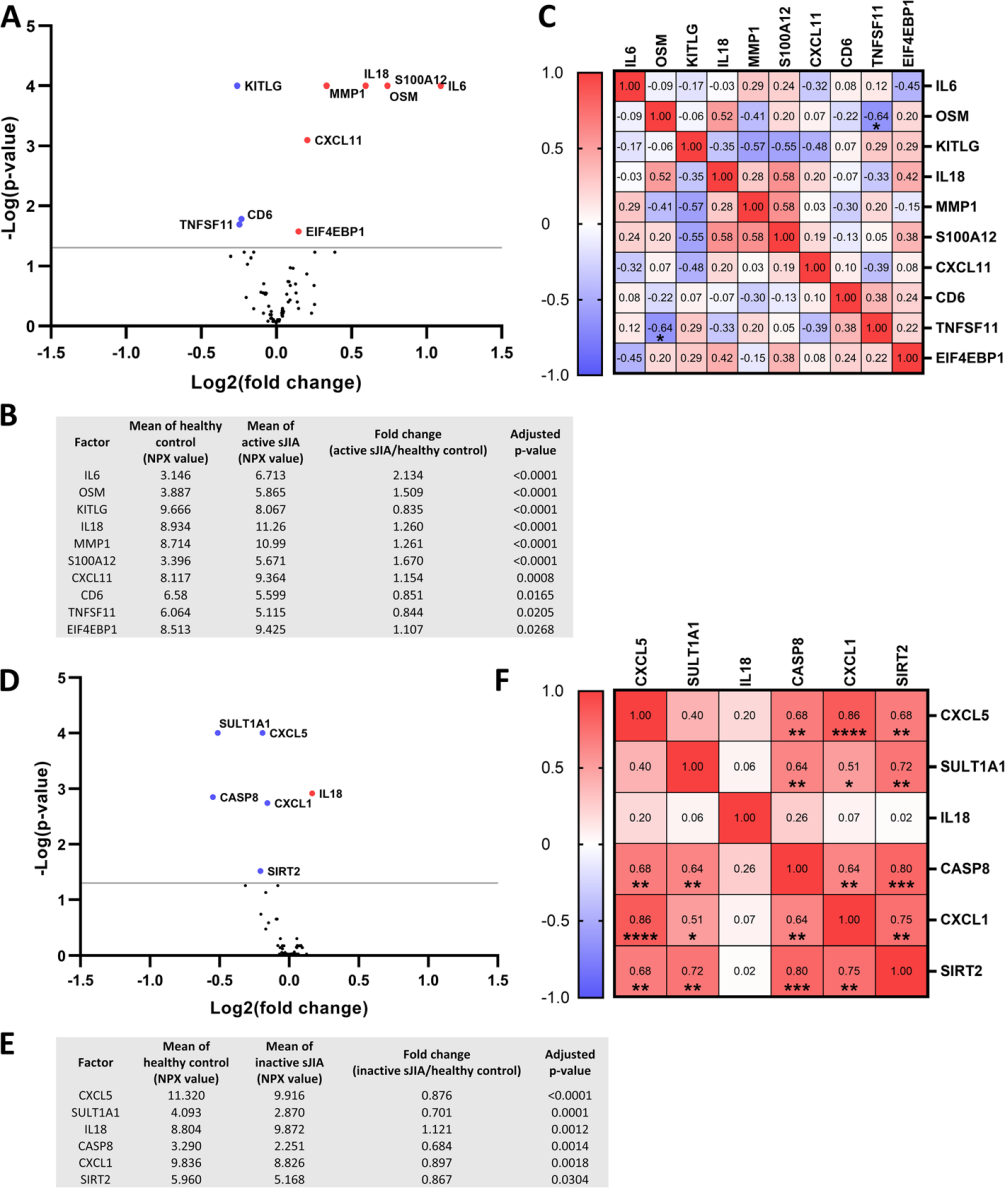
Comparative analyses of protein levels in active sJIA, inactive sJIA and matched-healthy controls. Volcano plot of biomarkers between (**A**) active sJIA and healthy controls, and (**D**) inactive sJIA and healthy controls. Dots with colors (blue representing lower levels in patients and red representing higher levels in patients as compared with controls) are significantly different between the compared groups (p < 0.05). **B and E** Summary of the significantly different proteins in the two comparison pairs. **C and F** Heat maps show correlation between disease-associated protein levels among sJIA patients. Statistics: **A, B, D** and **E** Two-way ANOVA with correction of multiple comparison by controlling the False Discovery Rate (FDR) of 5% via two-stage step-up method of Benjamini, Krieger and Yekutieli. **C** and **F**) Spearman correlation, * *p* < 0.05, ** *p* < 0.01, *** p < 0.001, **** *p* < 0.0001

To identify the proteins that could separate the inactive sJIA or the active sJIA group from the healthy control group, cross-sectional analyses were performed. Ten proteins were significantly different between active sJIA and healthy controls, in which seven protein were upregulated (IL6, OSM, IL18, MMP1, S100A12, CXCL11 and EIF4EBP1) while three were downregulated (KITLG, CD6 and TNFSF11) (Fig. 2A and B). Among the ten proteins, only OSM and TNFSF11 were negatively correlated with statistical significance (r = − 0.64, *p* = 0.028) (Fig. 2C). Levels of six proteins out of the 69 analyzed were significantly different between inactive sJIA and healthy controls (Fig. 2D and E): CASP8, CXCL1, CXCL5, SIRT2 and SULT1A1 showed lower levels in inactive sJIA, and these five proteins were positively correlated with statistical significance, especially CXCL1 and CXCL5 were highly positively correlated (r = 0.86, *p* < 0.0001). Only one protein showed higher level in inactive sJIA compared to healthy controls, namely IL18. Full data set of the cross-sectional analysis is presented in Supplementary Table S4.

### Paired analysis of protein levels in active and inactive sJIA identified proteins that distinguish sJIA activity

**Fig. 3:**
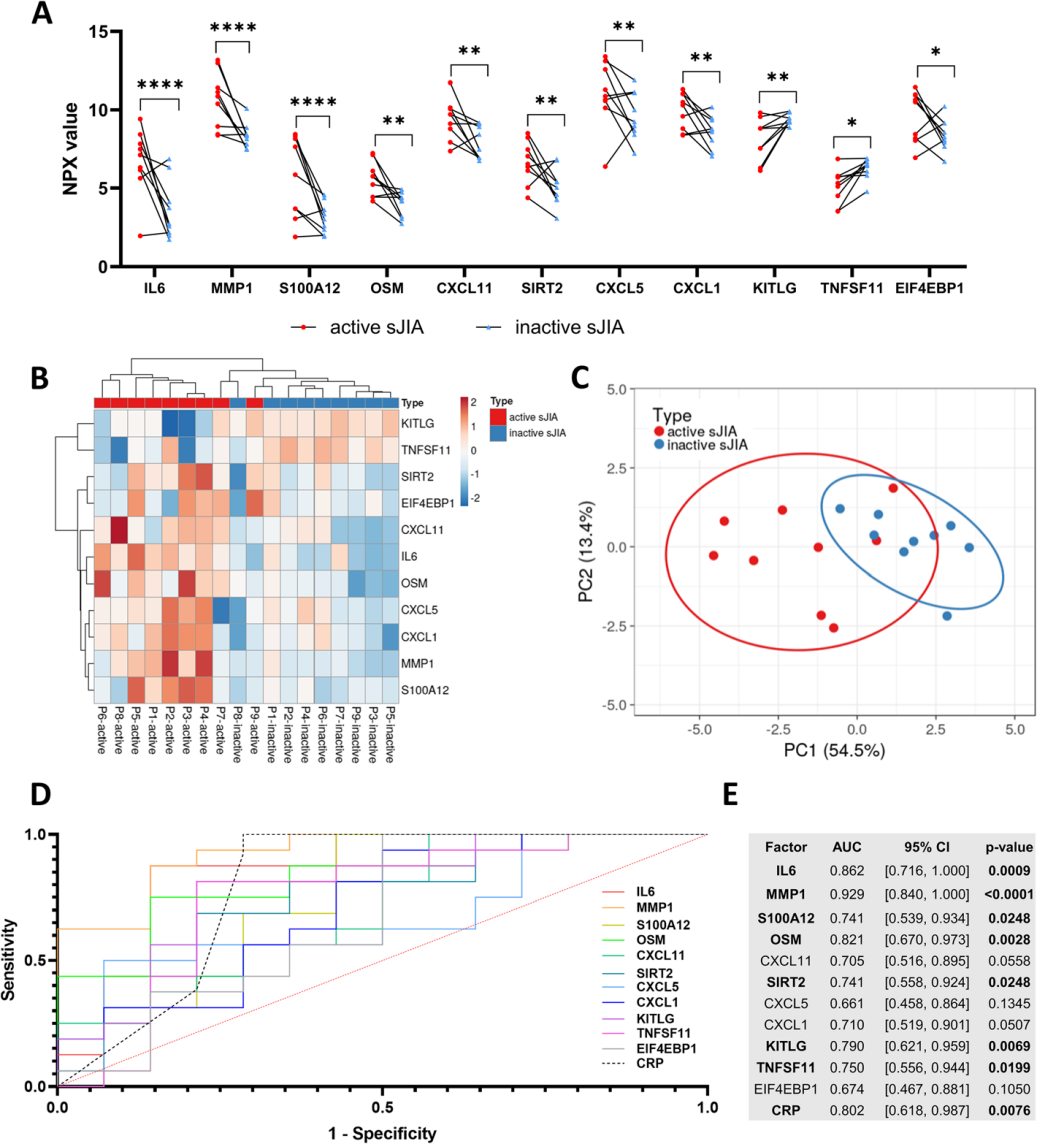
Paired analysis of active and inactive sJIA**. A** Comparison of protein levels in paired samples from nine patients during active and inactive disease phases. Each symbol represents one sample, and the lines link the paired samples. **B** Hierarchical clustering analysis shows the grouping of active and inactive sJIA based on the eleven significantly different proteins. In the heat map, rows were centered and unit variance scaling was applied to rows; both rows and columns were clustered using maximum distance and average linkage. **C** Principle component analysis of the three groups. The confidence level of the ellipses is 0.90. **D** Receiver operating characteristic (ROC) curves examining the predictive performance of the eleven proteins for distinguishing active sJIA (*n* = 14) in reference to inactive sJIA (*n* = 16) in the cross-sectional analysis. **E** Area under the curve (AUC) and corresponding 95% CI for each measure. Statistics: **A** two-way repeat-measurement ANOVA with correction of multiple comparison by controlling the False Discovery Rate (FDR) of 5% via two-stage step-up method of Benjamini, Krieger and Yekutieli, * p < 0.05, ** p < 0.01, **** p < 0.0001, (E) the 95% confidence interval were calculated by hybrid Wilson/Brown method

To further analyze whether any proteins could distinguish active and inactive sJIA, paired samples from nine patients obtained during active and inactive disease phases were investigated. 11 proteins had significantly different levels, with IL6, MMP1 and S100A12 having *p*-values lower than 0.0001 (Fig. 3A). These data were in agreement with the results in the cross-sectional analysis. Full data sets are presented in Supplementary Table S5. Based on the 11 differently expressed proteins rather than including all 69 proteins, active sJIA and inactive sJIA were better separated by HCA (Fig. 3B) and PCA (Fig. 3C).

The disease activity predictive performance of the 11 proteins was examined by enlarging the subject cohort from the nine-patient paired analysis to the 21-patient cross-sectional analysis. Receiver Operating Characteristic (ROC) curves for the 11 proteins in the classification of active and inactive sJIA are shown in Fig. 3D. Only IL6, MMP1, S100A12, OSM, SIRT2, KITLG and TNFSF11 showed statistically significant efficiency and were comparable to CRP (Fig. 3D and E).

### Cellular functions and canonical pathways implicated in active and inactive phases of sJIA

**Fig. 4:**
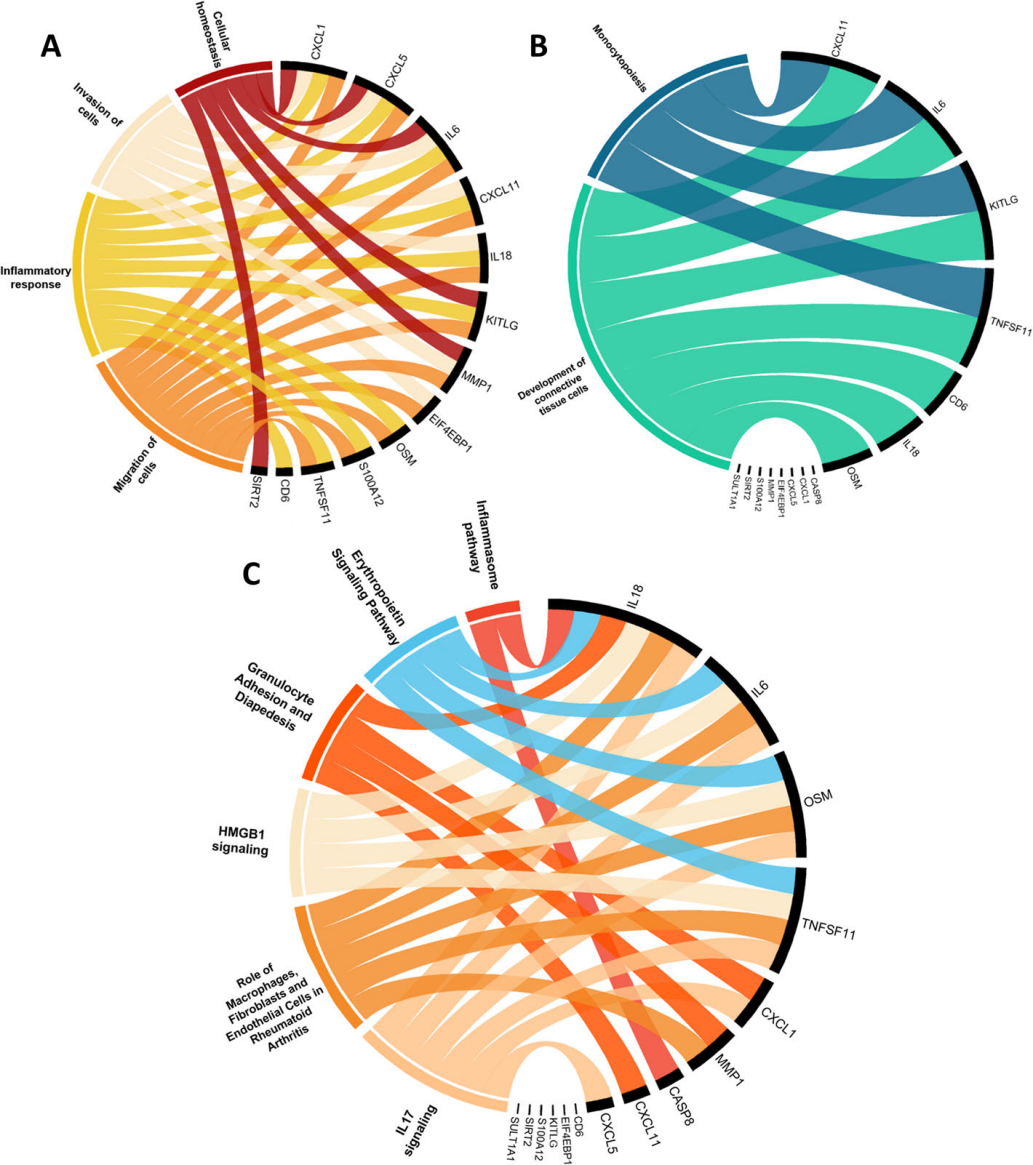
Cellular function and canonical pathways based on the proteins differentially expressed in sJIA. **T**he top cellular functions tended to be activated (**A**) and suppressed (**B**), as well as the top canonical pathways (**C**) were summarized in circular graphs. Except from Erythropoietin Signaling Pathway with negative z-score (− 2 < z-score < 0), all the pathways are with positive z-score (0 < z-score < 2)

To identify activated cell signaling pathways of importance for sJIA, proteins that were differentially expressed in the three comparison pairs, (i) active sJIA versus controls, (ii) inactive sJIA versus controls, and (iii) active sJIA versus inactive sJIA, were investigated using IPA to identify gene ontology groups and relevant canonical signaling pathways. However, no significant predication could be made by IPA, most likely due to the limited numbers of input factors (full data sets are presented in Supplementary Table S6-S11). Top cellular functions which tended to be activated or suppressed, as well as the top canonical pathways are summarized in circular graphs (Fig. 4).

Cell migration, cell invasion, cells homeostasis and inflammatory response appeared to be activated (Fig. 4A), while monocytopoiesis and development of connective tissues tended to be suppressed (Fig. 4B). IL-17 Signaling, HMGB1 Signaling, Granulocyte Adhesion and Diapedesis, Role of Macrophages, Fibroblasts and Endothelial Cells in Rheumatoid Arthritis, Inflammasome pathway were predicted with positive z-score (0 < z-score < 2), while Erythropoietin Signaling Pathway were predicted with negative z-score (− 2 < z-score < 0) (Fig. 4C).

### Levels of HMGB1 were higher in active sJIA than inactive sJIA, with significant disease activity prediction efficiency

**Fig. 5:**
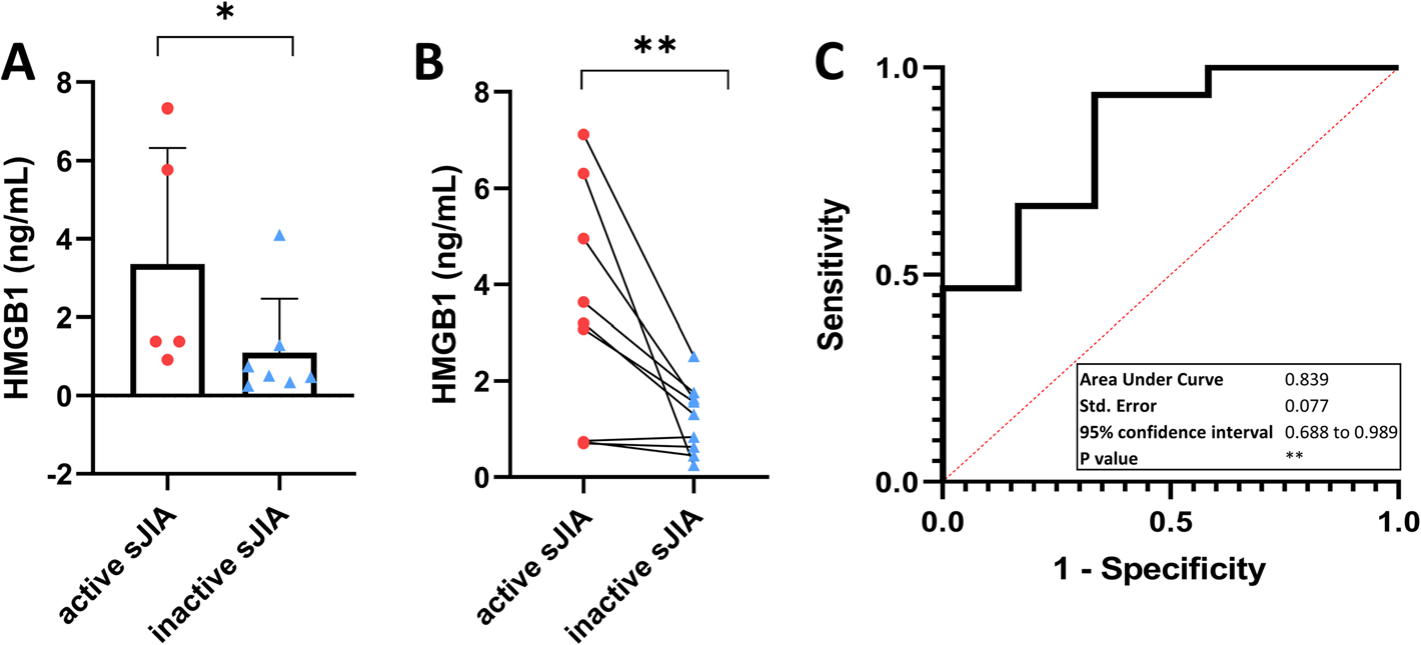
HMGB1 levels in plasma samples obtained during active and inactive sJIA. **A** Cross sectional analysis of HMGB1 levels in active and inactive sJIA patients. **B** Paired analysis of HMGB1 levels during active and inactive disease phases. **C** Receiver operator characteristics (ROC) analysis of HMGB1 concentration in the plasma of sJIA. Statistics: **A** Mann-Whitney U test, (**B**) Wilcoxon matched-pairs signed rank test, * p < 0.05, ** p < 0.01, (**C**) the 95% confidence interval were calculated by hybrid Wilson/Brown method

Compared with healthy controls, HMGB1 signaling tended to be upregulated in active sJIA (Fig. 4C). HMGB1 is a damage associated molecular pattern (DAMP), which has been studied in a series of inflammatory diseases, including JIA. Highly increased levels of HMGB1 have also been reported during MAS [16]. Since HMGB1 was not included in the PEA inflammation panel, HMGB1 levels were determined by ELISA. The active sJIA group had significantly higher HMGB1 levels than the inactive sJIA group (Fig. 5A). In paired analysis, eight of the nine patients had higher HMGB1 levels when in active disease phase, one patient had a slightly higher HMGB1 level in the inactive phase (0.84 ng/mL in inactive and 0.76 ng/mL in active) (Fig. 5B). The ROC analysis (AUC = 0.839, 95% confidence interval 0.688–0.989) confirmed the ability of HMGB1 to distinguish active and inactive sJIA (Fig. 5C).

## Discussion

We set out to define inflammatory mediators and pathways involved in sJIA and to define whether mediators and pathways differed during active and inactive disease phases. Our aim was to identify biomarkers that could aid and improve disease diagnosis and to reveal underlying disease mechanisms and how they may contribute to sJIA flares and remission. By taking both a cross-sectional and paired analysis approach when immunoprofiling a clinically well-described sJIA cohort, we have been able to identify sets of mediators with different plasma levels in active and inactive sJIA patients compared to healthy controls, and a set of mediators differing between active and inactive sJIA phases. As evidence suggests that circulating plasma levels of cytokines are influenced by age in healthy children [17–19], we initially analyzed if the pattern of the analyzed proteins showed an age-associated decrease in levels using sixty samples from healthy children at our disposal. The results clearly emphasize the importance of age and sex matched control groups in pediatric clinical studies (Supplementary Fig. S2 and Supplementary Table S2). It also made us select thirty age- and sex matched control samples rather than using the larger set of available samples.

Comparing to healthy controls, levels of ten proteins were significantly different in active sJIA. Highest difference in levels were defined for IL6, S100A12, IL18 and OSM. sJIA is based on abnormalities in the innate immune system leading to activation of immunocompetent cells, resulting in the release of proinflammatory IL6, IL18 and S100 proteins [20–22]. OSM belongs to the IL6 family. Monocyte and macrophages, a primary source of OSM, release OSM upon stimulation with agents such as ligands of Toll-like receptors [23].

MMP1 is an interstitial collagenase that degrades type II collagen in cartilage, and elevated levels of MMP1 are observed in arthritic tissues [24]. Indeed, we also found higher levels of MMP1 in synovial fluid compared to paired plasma samples in oligo-JIA patients (data unpublished). There has been no study analyzing plasma levels of MMP1 in sJIA. As most cells in the body can produce MMPs, the elevated MMP1 levels recorded could either be released from damaged tissues or released by activated peripheral immunocompetent cells.

CXCL11 is an IFNγ-induced chemokine, eliciting chemotactic activity in interleukin-activated T cells. Higher levels of CXCL11 in MAS than in active sJIA without MAS has been reported, with CXCL11 levels positively correlated with Ferritin levels [25].

We found KITLG (Stem Cell Factor) to be significantly downregulated in active sJIA. KITLG is the ligand of receptor-type protein-tyrosine kinase KIT, regulating apoptosis, cell proliferation, differentiation and migration. Thus, KITLG might have multiple roles in sJIA. Similarly, a study reported KITLG to be downregulated in Kawasaki disease and suggested that KITLG might play an essential role in inflammatory syndromes [26]. There are no previous reports of the role of KITLG in pediatric arthritis.

Membrane-bound CD6 (mCD6) regulates T cell activity, and deficiency of mCD6 reduced both T cell activity and cytokine production in collagen-induced arthritis [27, 28]. Soluble CD6 (sCD6) measured in this study is regarded as a decoy receptor. It might be speculated that diminished expression of sCD6 in active sJIA contributes to the increased disease activity.

We determined lower levels of TNFSF11 in active sJIA than inactive sJIA. TNFSF11 (receptor activator of nuclear factor-κB ligand, RANKL) exerts its biologic effects by binding to RANK and inducing osteoclast differentiation and activation. There are contradictory data on the levels of TNFSF11 in sera of JIA compared to healthy controls [29, 30], while no study has previously been performed on sJIA. It is possible that increased binding of TNFSF11 to its receptor results in a lower plasma level of TNFSF11. In addition to its osteotropic effects, TNFSF11 also activates the anti-apoptotic kinase AKT/PKB through a signaling complex indicating that TNFSF11 may have a role in the regulation of apoptosis [31]. Therefore, the lower plasma level of TNFSF11 may reveal the immune dysregulation in active sJIA.

More proteins expressed in significantly lower levels than in higher levels were defined when comparing inactive sJIA to controls. The highest difference was defined for CXCL5 and SULT1A1. CXCL1 and CXCL5 are neutrophil chemoattractants. SULT1A1 is a sulfotransferase. SULT1A1 has been reported to be upregulated in sera of ulcerative colitis patients [32], while no studies on the role of SULT1A1 in arthritis have been reported.

CASP8 is known as an initiator of apoptosis and a suppressor of necroptosis. However, the role of CASP8 in JIA has never been explored. Our finding of lower levels of CASP8 in inactive sJIA than in healthy controls may reflect the cell death pattern during disease resolution.

SIRT2 is an NAD+ (nicotinamide adenine dinucleotide)-dependent deacetylase. SIRT2 was reported to physically interact with and regulate NF-κB activation by deacetylating the p65 subunit [33]. In collagen-induced arthritis, deficiency of *Sirt2* resulted in a more severe arthritic phenotype [34]. We found SIRT2 to be lower in inactive sJIA than active sJIA (paired analysis) and lower in inactive sJIA than in healthy controls (cross-sectional analysis), which may reveal a distinct mechanism during disease inactivation.

Interestingly, except from IL18, no biomarker was a shared hit between the two comparison pairs (active sJIA versus healthy control and inactive sJIA versus healthy control), indicating different disease mechanisms during active and inactive sJIA. Lower levels of certain inflammatory mediators in inactive sJIA compared to controls suggest that suppressive mechanisms are activated during disease resolution. Loss of such down-regulatory mechanisms could contribute to disease flares.

The paired analysis between active and inactive sJIA followed by HCA showed two active sJIA samples clustered into the inactive sJIA group. These patients’ medical records revealed that they had lower CRP levels or milder symptoms than other patients with active sJIA, which may explain the clustering pattern observed. Despite diverse medication regimes, the paired analysis showed a general change in the levels of multiple inflammatory proteins, though only IL6, MMP1, S100A12, OSM, SIRT2, KITLG and TNFSF11 showed satisfying disease activity predictive efficiency when the cohort was enlarged. Together, this group of proteins could potentially be applied to stratify and predict sJIA activity. Although the ROC curves provided important information regarding the synergetic effects of combining markers, the direct clinical application remains limited due to the small cohort in this study; therefore, further validation is needed.

In paired analysis, we found the level of IL18 was not significantly difference between active and inactive phase of sJIA patients. Compared to healthy controls, IL18 remained elevated in inactive sJIA. This might be explained by the patients included in this study had chronic sJIA, previously reported to show sustained elevated serum IL18 levels even during inactive phases [35]. Thus, IL18 may be less suited as an activity predictor than IL6, MMP1 or S100A12.

To further clarify sJIA pathogenesis and to reveal the interactions between the altered biomarkers defined in our study, we performed pathway analysis via IPA. Based on the limited number of input protein, no significant canonical pathway was predicted, although several cellular functions and pathways tended to be activated or suppressed. Among the top-ranked pathways was HMGB1 signaling pathway, though HMGB1 was not included in the PEA panel used. Similar to S100 proteins, HMGB1 is a DAMP and has been investigated in autoimmune diseases, including RA [36], systemic lupus erythematosus (SLE) [37] and juvenile SLE [38]. We have previously reported significantly increased HMGB1 levels in JIA synovial fluid [39]. Recently, Xu et al. [40] reported that serum HMGB1 levels at the first visit were significantly elevated in sJIA compared with other JIA subgroups. We found significantly higher HMGB1 levels in active than in inactive phases, and confirmed HMGB1’s predictive ability of sJIA activity by ROC analysis. HMGB1 could thus be a potential driver of sJIA and a possible therapeutic target.

The limitations of this study include the relatively small patient cohort and their different medication regimes. Medications are likely to affect the immune activity and may interfere with plasma levels of inflammatory protein. However, in actual clinical settings, patients are often with various symptoms and complicated medical history. Defining biomarkers with diagnosis and prognosis efficacy regardless of medication regimes could be more practically significant. Additionally, though the Olink inflammatory panel involved representative inflammatory-related proteins, several proteins, such as IL1β and S100A8/9, are not included in the panel and hence not in our analysis.

## Conclusion

By applying a high-throughput and multiplex immunoassay, we have defined inflammatory proteins that differ significantly between inactive sJIA patients and healthy controls, between active sJIA patients and healthy controls, and between inactive and active sJIA patients. In this explorative study, we not only confirmed the previously reported sJIA biomarkers IL6, IL18, S100A12 and OSM, but also identified new potential biomarkers CASP8, CCL23, CD6, CXCL1, CXCL11, CXCL5, EIF4EBP1, KITLG, MMP1, OSM, SIRT2, SULT1A1 and TNFSF11 that characterized sJIA patients in active and inactive phases. Further studies of the pathogenic features of the newly revealed biomarkers and their potential use as diagnostic tools and as new targets for therapy development are highly warranted.

## Supplementary Information

**Additional file 1.** Supplementary methods, tables and figures

## Data Availability

The datasets supporting the conclusions of this article are included within the article and its additional files.

## Abbreviations

AUC: Area under the curve
CASP8: Caspase 8
CCL23: C-C motif chemokine ligand 23
CRP: C-reactive protein
CXCL1: C-X-C motif chemokine ligand 1
CXCL11: C-X-C motif chemokine ligand 11
CXCL5: C-X-C motif chemokine ligand 5
DAMP: Damage associated molecular pattern
EIF4EBP1: Eukaryotic translation initiation factor 4E binding protein 1
ELISA: Enzyme-Linked ImmunoSorbent Assay
ESR: Erythrocyte sedimentation rate
FDR: False Discovery Rate
FGF23: Fibroblast growth factor 23
HCA: Hierarchical clustering analysis
HMGB1: High Mobility Group Box 1
IL: Interleukin
INFγ: Interferon gamma
IPA: Ingenuity Pathway Analysis
JIA: Juvenile idiopathic arthritis
KITLG: C-Kit ligand
MAS: Macrophage activation syndrome
mCD6: Membrane-bound CD6
MMP: Matrix Metalloproteinase
NPX: Normalized protein expression
OSM: Oncostatin M
PCA: Principal component analysis
PEA: Proximity Extension Assay
PLAU: Urolinase-type plasminoen activator
RA: Rheumatoid arthritis
RF: Random Forest
ROC: Receiver Operating Characteristic
S100A12: S100 calcium binding protein A12
sCD6: Soluble CD6
SIRT2: Sirtuin 2
sJIA: Systemic juvenile idiopathic arthritis
SLE: Systemic lupus erythematosus
SULT1A1: Sulfotransferase family 1A member 1
TNFB: Lymphotoxin alpha
TNFSF11: TNF superfamily member 11
