# Supplementary material for: Immunoprofiling of active and inactive systemic juvenile idiopathic arthritis reveals distinct biomarkers: a single-center study
Source: Pediatr Rheumatol Online J. 2021 Dec 28;19:173. doi: 10.1186/s12969-021-00660-9 (PMC8713412; doi:10.1186/s12969-021-00660-9)
Supplement: Supplementary file 1 — Additional file 1. Supplementary methods, tables and figures [file 12969_2021_660_MOESM1_ESM.pdf]

Immunoprofiling of Active and Inactive Systemic Juvenile Idiopathic Arthritis Reveals Distinct Biomarkers: A Single-Center Study

Supplementary documents

Contents

Supplementary methods 3

1. Assay methodology 3

1.1 Overview of Proximity Extension Assay (PEA) 3

1.2 Targets of the Olink Inflammation panel 3

1.3 Overview of PEA protocol 3

1.4 Data presentation 4

2. Quality control 4

2.1 Protocol for internal quality control 4

2.2 Protocol for marker quality control 4

2.3 Batch correction 4

3. Statistical analysis 5

3.1 General statistical analysis methods 5

3.2 Receiver operating characteristic (ROC) curve 5

Supplementary Tables 6

Supplementary Table S1. List of all 92 biomarkers analyzed in Olink inflammatory panel. . 6

Supplementary Table S2. Average NPX values of each analyzed proteins in healthy control groups with different age. 8

Supplementary Table S3. Random forest analysis identified the proteins' contribution to the separation of the three groups. 9

Supplementary Table S4. Detailed fold change and p-values between the cross-sectional comparisons. 10

Supplementary Table S5. Detailed fold change and p-Values between active and inactive sJIA paired analysis. 11

Supplementary Table S6. Top cellular functions results from comparison between active sJIA and healthy controls. 12

Supplementary Table S7. Top cellular functions results from comparison between inactive sJIA and healthy controls. 12

Supplementary Table S8. Top cellular functions results from comparison between active sJIA and inactive sJIA. 12

Supplementary Table S9. Top canonical pathways results from comparison between active sJIA and healthy controls. 13
Supplementary Table S10. Top canonical pathways results from comparison between inactive sJIA and healthy controls. 13
Supplementary Table S11. Top canonical pathways results from comparison between active sJIA and inactive sJIA. 13
Supplementary Figures 14
Supplementary Fig. S1. Normalization of two plasma datasets from different inflammation panel versions. 14
Supplementary Fig. S2. Age is a major confounding factor and gender also matters 15
Supplementary Fig. S3. Illustration of analysis settings in this study. 16
Supplementary Fig. S4. Distribution of the different subgroups based on 69 detected inflammation-associated proteins. 17
References 18

Supplementary methods

1. Assay methodology

1.1 Overview of Proximity Extension Assay (PEA)

The Olink reagents are based on the Proximity Extension Assay (PEA) technology [1], where 92 oligonucleotide labeled antibody probe pairs are allowed to bind to their respective target proteins, if present in the sample. A PCR reporter sequence is formed by a proximity-dependent DNA polymerization event. This is then amplified, and subsequently detected and quantified using real-time PCR. The assay is performed in a homogeneous 96-well format without any need for washing steps.

1.2 Targets of the Olink Inflammation panel

Inflammation is a biological process of central importance for biomedical research and is now believed to be a key underlying factor for the pathophysiology of a wide range of diseases, from “classical” inflammatory conditions (for example, inflammatory bowel disease and dermatological diseases) to cardiovascular disease and cancer. Olink Inflammation panel provides a high-throughput, multiplex immunoassay enabling analysis of 92 inflammation-related protein biomarkers across 96 samples simultaneously.

This represents the most extensive panel available on the market for proteins associated with inflammatory diseases and related biological processes, enabling investigation of protein signatures with high efficiency and robustness and accelerates the speed of finding new and relevant human protein biomarkers related to inflammation. The panel is compiled to detect a selection of already established as well as exploratory biomarkers within the inflammation research field. The content of the panel has been designed in close collaboration with experts within various disease areas such as rheumatoid arthritis, Crohn’s disease, ulcerative colitis, neuro inflammation, and respiratory diseases, and it is well suited to discerning protein profiles in clinically relevant samples.

The validation data of Olink inflammation panel are presented on https://www.olink.com/content/uploads/2019/04/Olink-Inflammation-Validation-Data-v3.0.pdf

1.3 Overview of PEA protocol

One microliter plasma from each sample was mixed with three microliters of an incubation mix on a 96-well plate and incubated overnight at temperature of two-to-four degrees Celsius. An extension mix including PCR polymerase was added to each well then placed into a thermal cycler. In the detection phase, 2.8 microliters from each well were then mixed with 7.2 microliters of a detection mix and placed on a 96.96 Dynamic Array Integrated Fluidic Circuit (IFC) chip along with the corresponding ninetytwo primer pairs. The chip was then ran through the Fluidigm BioMark reader using standard protocol provided by the supplier. Samples were run in singlets in parallel with both blanks and inter-plate/batch controls.

Details regarding both assay limitations, validations, and protocols may be obtained from the Olink supplier (http://www.olink.com).

1.4 Data presentation

Normalized Protein eXpression values (NPX) are delivered for all proteins. NPX is on a log2 scale which means that an increase in one NPX corresponds to a doubling of the concentration. NPX gives a relative quantification, as the information shown in https://www.olink.com/question/what-is-npx/. Thus, even if two different proteins have the same NPX values, their actual concentration may differ. NPX is generated by a combination of the Fluidigm multiplex qPCR system and Olink's NPX manager. Normalization is performed to minimize both intra- and inter-assay variation. For more information about the normalization steps, see http://www.olink.com/question/how-is-the-data-pre-processed/.

Olink results show excellent parallellism when performing a dilution series and displays excellent linearity. Olink results are more consistent with MSD results, than any of MSD or Olink versus Luminex. Olink data is therefore largely consistent with well-established low- to mid-plex methods, and offers a much broader, scalable solution with unmatched specificity at high multiplexing levels, with uniquely low sample consumption. More details could be found in Olink white paper https://f.hubspotusercontent40.net/hubfs/7074596/White%20papers/olink-white-paper_a-comparative-study-across-multiple-platforms_v1.2.pdf?hsCtaTracking=a0a4300d-1b10-4b11-8bbc-8831f68806cb%7C06791960-6d4c-4085-8f94-5788b9daf23b

2. Quality control

2.1 Protocol for internal quality control

Assay includes four internal controls aimed at monitoring performance and reliability during the processes of immuno-reaction, extension, and amplification. This includes two incubation control proteins, phycoerythrin (PE) and green fluorescent protein (GFP); a single extension control consisting of IgG antibodies conjugated with matching oligo pair; and a detection control with a synthetic double-stranded DNA. Overall quality of each plate was evaluated by the standard deviation of internal controls with a threshold below 0.2 NPX. Samples with high variability (above ± 0.3 NPX) from the median of the plate were flagged for removal. In addition, external controls including inter-plate, negative, and interbatch controls were used to determine potential issues in assay quality, high variability, background noise, and errors in handling along with general normalization of batch effects.

2.2 Protocol for marker quality control

Markers were assessed based on their call rate (i.e. proportion of samples with measurable concentrations above the limit of detection) and variability. Of the 92 proteins analyzed, 71 proteins had a call rate above 80%, all undetermined measurements were due to concentrations below the lower limit of detection (LLOD). Twenty-one plasma proteins consisting of MCP-3, GDNF, IL-20RA, IL-2RB, IL-1 alpha, IL2, IL-17C, TSLP, SLAMF1, FGF-5, IL-22 RA1, Beta-NGF, IL-24, IL13, ARTN, IL-20, IL33, IL4, LIF, NRTN and IL5 had a low call rate below 20% and were excluded in the analysis. Eight plasma proteins consisting of IL17A, IL10RA, IL15RA, CASP8, IL6, FGF23, CCL28 and SULT1A1 had a low call rate between 20-80% and were included in certain analyses but were interpreted with precaution. Additional details of the approximate call rate for detected proteins are listed in Supplementary Table S1.

2.3 Batch correction

The plasma samples included in this study were run in two versions of Olink Inflammation panel, separately, with eight bridging samples, for normalization between the two runs. The validity of normalization was controlled by checking absence of grouping according to panel

version in principle component analysis (PCA) (Supplementary Fig. S1). IFNγ and TNF had to be excluded from the analysis due to a change in antibody pairs between the two versions.

In summary, 69 proteins in plasma passed quality control and were included for the analysis.

3. Statistical analysis

3.1 General statistical analysis methods

Protein measures were statistically analyzed with the default log base-two transformed protein levels (i.e. one NPX unit increase equals a doubling in concentration).

1) In the cross-sectional analysis, ordinary two-way ANOVA was performed on active sJIA (n=14), inactive sJIA (n=16) and healthy controls (n=30) (Fig. 1).
2) Two-way ANOVA was performed on active sJIA (n=14) versus healthy controls (n=28) and on inactive sJIA (n=16) versus healthy controls (n=32), separately (Fig. 2).

(In Fig. 1 and 2, different number of healthy controls was used to compare with different groups of patients, to always keep the control group age- and gender-match to patients.)

3) In the paired analysis, two-way repeat-measurement ANOVA was performed on paired active sJIA (n=9) and inactive sJIA (n=9) samples from 9 patients (Fig. 3).
4) Mann-Whitney U test was performed on the HMGB1 levels from active sJIA (n=5) verus inactive sJIA (n=7) (Fig. 5A). Wilcoxon matched-pairs signed rank test was was performed on paired active sJIA (n=9) and inactive sJIA (n=9) samples from 9 patients (Fig. 5B).

All the above analysis was corrected for multiple comparison by controlling the False Discovery Rate (FDR) of 5% via two-stage step-up method of Benjamini, Krieger and Yekutieli, adjusted p-values less than 0.05 were regarded as significant. GraphPad Prism version 8.4.3 (San Diego, CA, USA) was used for the statistical analysis.

3.2 Receiver operating characteristic (ROC) curve

ROC curves were used to compare the efficacy of the top significant factors from active-inactive sJIA paired analysis and HMGB1 for classifying disease activity in unpaired active and inactive sJIA groups (Fig. 3D and 5C). Predictively of candidate markers were considered individually and in combination through a logistic regression model run by IBM SPSS Statistics Version 26.0.0.0. Although the ROC curves provided important information regarding the synergetic effects of combining markers, direct clinical application remains limited due to the small cohort in this study, therefore further validation is needed.

Supplementary Tables

Supplementary Table S1. List of all 92 biomarkers analyzed in Olink inflammatory panel.
Call rate represents the proportion of samples with measurable concentrations above the limit of detection. Proteins with a low call rate below 20% were excluded from further the analysis

ID (Olink ID if different)	Entrez Gene Name	Type(s)	Call rate (%)
ADA	adenosine deaminase	enzyme	>80%
ARTN	artemin	growth factor	<20%
AXIN1	axin 1	other	>80%
CASP8	caspase 8	peptidase	20-80%
CCL11	C-C motif chemokine ligand 11	cytokine	>80%
CCL13 (MCP-4)	C-C motif chemokine ligand 13	cytokine	>80%
CCL19	C-C motif chemokine ligand 19	cytokine	>80%
CCL2 (MCP-1)	C-C motif chemokine ligand 2	cytokine	>80%
CCL20	C-C motif chemokine ligand 20	cytokine	>80%
CCL23	C-C motif chemokine ligand 23	cytokine	>80%
CCL25	C-C motif chemokine ligand 25	cytokine	>80%
CCL28	C-C motif chemokine ligand 28	cytokine	20-80%
CCL3	C-C motif chemokine ligand 3	cytokine	>80%
CCL4	C-C motif chemokine ligand 4	cytokine	>80%
CCL7 (MCP-3)	C-C motif chemokine ligand 7	cytokine	<20%
CCL8 (MCP-2)	C-C motif chemokine ligand 8	cytokine	>80%
CD244	CD244 molecule	transmembrane receptor	>80%
CD274 (PD-L1)	CD274 molecule	enzyme	>80%
CD40	CD40 molecule	transmembrane receptor	>80%
CD5	CD5 molecule	transmembrane receptor	>80%
CD6	CD6 molecule	transmembrane receptor	>80%
CD8A	CD8a molecule	other	>80%
CDCP1	CUB domain containing protein 1	other	>80%
KITLG (SCF)	c-Kit ligand	growth factor	>80%
CSF1	colony stimulating factor 1	cytokine	>80%
CST5	cystatin D	other	>80%
CX3CL1	C-X3-C motif chemokine ligand 1	cytokine	>80%
CXCL1	C-X-C motif chemokine ligand 1	cytokine	>80%
CXCL10	C-X-C motif chemokine ligand 10	cytokine	>80%
CXCL11	C-X-C motif chemokine ligand 11	cytokine	>80%
CXCL5	C-X-C motif chemokine ligand 5	cytokine	>80%
CXCL6	C-X-C motif chemokine ligand 6	cytokine	>80%
CXCL9	C-X-C motif chemokine ligand 9	cytokine	>80%
DNER	delta/notch like EGF repeat containing	transmembrane receptor	>80%
EIF4EBP1 (4E-BP1)	eukaryotic translation initiation factor 4E binding protein 1	translation regulator	>80%
FGF19	fibroblast growth factor 19	growth factor	>80%
FGF21	fibroblast growth factor 21	growth factor	>80%
FGF23	fibroblast growth factor 23	growth factor	20-80%
FGF5	fibroblast growth factor 5	growth factor	<20%
FLT3LG (Flt3L)	fms related tyrosine kinase 3 ligand	cytokine	>80%
GDNF	glial cell derived neurotrophic factor	growth factor	<20%
HGF	hepatocyte growth factor	growth factor	>80%
IL10	interleukin 10	cytokine	20-80%
IL10RA	interleukin 10 receptor subunit alpha	transmembrane receptor	20-80%
IL10RB	interleukin 10 receptor subunit beta	transmembrane receptor	>80%
IL12B	interleukin 12B	cytokine	>80%
IL13	interleukin 13	cytokine	<20%
IL15RA	interleukin 15 receptor subunit alpha	transmembrane receptor	>80%
IL17A	interleukin 17A	cytokine	20-80%
IL17C	interleukin 17C	cytokine	<20%
IL18	interleukin 18	cytokine	>80%
IL18R1	interleukin 18 receptor 1	transmembrane receptor	>80%
IL1A	interleukin 1 alpha	Extracellular Space	>80%
IL2	interleukin 2	Extracellular Space	>80%
IL20	interleukin 20	Extracellular Space	<20%
IL20RA	interleukin 20 receptor subunit alpha	Plasma Membrane	<20%
IL22RA1	interleukin 22 receptor subunit alpha 1	Plasma Membrane	<20%
IL24	interleukin 24	Extracellular Space	<20%
IL2RB	interleukin 2 receptor subunit beta	Plasma Membrane	<20%
IL33	interleukin 33	Extracellular Space	<20%
IL4	interleukin 4	Extracellular Space	<20%
IL5	interleukin 5	Extracellular Space	<20%
IL6	interleukin 6	Extracellular Space	20-80%
IL7	interleukin 7	Extracellular Space	20-80%
IL8	C-X-C motif chemokine ligand 8	Extracellular Space	>80%
INFγ	Interferon gamma	Extracellular Space	NA*
LIF	LIF interleukin 6 family cytokine	Extracellular Space	<20%
LIFR	LIF receptor subunit alpha	Plasma Membrane	>80%

MMP1	matrix metallopeptidase 1	Extracellular Space	>80%
MMP10	matrix metallopeptidase 10	Extracellular Space	>80%
NGF (Beta-NGF)	nerve growth factor	Extracellular Space	<20%
NRTN	neurturin	Extracellular Space	<20%
OPG	TNF receptor superfamily member 11b	Plasma Membrane	>80%
OSM	oncostatin M	Extracellular Space	>80%
PLAU (uPA)	Urolinase-type plasminoen activator	Extracellular Space	>80%
S100A12 (EN-RAGE)	S100 calcium binding protein A12	Cytoplasm	>80%
SIRT2	sirtuin 2	Nucleus	20-80%
SLAMF1	signaling lymphocytic activation molecule family member 1	Plasma Membrane	<20%
NTF3 (NT-3)	Neurotrophin 3	Extracellular Space	20-80%
STAMBP	STAM binding protein	Nucleus	>80%
SULT1A1 (ST1A1)	sulfotransferase family 1A member 1	Cytoplasm	20-80%
TGFA	transforming growth factor alpha	Extracellular Space	>80%
TGFB1 (LAP TGF-beta-1)	transforming growth factor beta 1	Extracellular Space	>80%
TNF	Tumor necrosis factor	Extracellular Space	NA*
TNFB	lymphotoxin alpha	Extracellular Space	>80%
TNFRSF9	TNF receptor superfamily member 9	Plasma Membrane	>80%
TNFSF10 (TRAIL)	TNF superfamily member 10	Extracellular Space	>80%
TNFSF11 (TRANCE)	TNF superfamily member 11	Extracellular Space	>80%
TNFSF12 (TEWAK)	TNF superfamily member 12	Extracellular Space	>80%
TNFSF14	TNF superfamily member 14	Extracellular Space	>80%
TSLP	thymic stromal lymphopoietin	Extracellular Space	<20%
VEGFA	vascular endothelial growth factor A	Extracellular Space	>80%

* The patient plasma samples were run in two versions of Olink Inflammation panel with eight bridging samples, for normalization between the two runs. The validity of normalization was controlled by checking absence of grouping according to panel version in principle omponent analysis (PCA) (Supplementary Fig. 1). IFNγ and TNF had to be excluded from the analysis due to a change in antibody pairs between the two versions. Therefore, 90 proteins were included in the analysis.

Supplementary Table S2. Average NPX values of each analyzed proteins in healthy control groups with different age. The proteins were mainly different between 4-year-old and 12-year-old groups. Statistics: Two-way ANOVA with correction of multiple comparison by controlling the False Discovery Rate (FDR) of 5% via two-stage step-up method of Benjamini, Krieger and Yekutieli.

	Mean of 4-year-old (NPX value)	Mean of 8-year-old (NPX value)	Mean of 12-year-old (NPX value)	4-year-old v.s. 8-year-old Adjust p-values	4-year-old v.s. 12-year-old Adjust p-values	8-year-old v.s. 12-year-old Adjust p-values
IL8	5.878	5.457	5.009	0.338	0.006	0.425
VEGFA	10.750	10.510	10.260	0.525	0.026	0.586
CDCP1	3.036	2.975	2.969	0.785	0.436	>0.999
CD244	7.639	7.281	7.225	0.250	0.056	0.964
IL7	4.420	3.640	3.406	0.250	0.011	0.805
OPG	10.010	9.841	9.821	0.250	0.056	0.964
TGFB1	7.893	7.604	7.230	0.554	0.029	0.539
PLAU	10.300	10.080	10.200	0.100	0.255	0.464
IL6	2.978	3.229	3.287	0.516	0.123	0.964
CCL2	10.960	10.780	10.870	0.370	0.333	0.789
IL17A	2.353	1.860	1.683	0.101	0.005	0.586
CXCL11	8.844	8.678	8.122	0.863	0.082	0.586
AXIN1	5.517	4.520	4.111	0.359	0.031	0.789
TNFSF10	8.300	8.182	8.305	0.395	0.662	0.482
CXCL9	6.992	6.883	6.477	0.785	0.029	0.425
CST5	5.753	5.532	5.416	0.395	0.029	0.789
OSM	4.290	4.435	3.555	0.857	0.083	0.202
CXCL1	10.39	9.315	9.376	0.111	0.008	0.964
CCL4	6.611	6.240	5.908	0.370	0.006	0.569
CD6	7.000	6.566	6.182	0.250	0.006	0.464
KITLG	9.532	9.539	9.666	>0.999	0.285	0.425
IL18	9.377	9.108	8.851	0.395	0.029	0.586
TGFA	3.751	3.746	3.724	>0.999	0.564	0.964
CCL13	13.800	13.210	13.060	0.370	0.050	0.915
CCL11	6.511	6.372	6.217	0.584	0.031	0.730
TNFSF14	4.791	4.797	3.848	>0.999	0.010	0.166
FGF23	2.850	2.906	2.946	0.617	0.198	0.886
IL10RA	1.268	0.931	1.125	0.366	0.436	0.482
MMP1	8.806	9.351	8.595	0.410	0.449	0.425
LIFR	3.655	3.707	3.764	0.672	0.209	0.805
FGF21	2.618	2.585	3.274	>0.999	0.102	0.464
CCL19	9.945	9.686	9.661	0.315	0.043	0.964
IL15RA	1.452	1.206	1.306	0.064	0.042	0.586
IL10RB	5.998	5.923	6.156	0.672	0.096	0.202
IL18R1	8.322	8.305	8.345	>0.999	0.602	0.948
CD274	6.862	6.669	6.765	0.315	0.375	0.789
CXCL5	12.120	10.730	10.770	0.156	0.018	>0.999
TNFSF11	6.309	5.881	6.172	0.090	0.301	0.425
HGF	8.763	8.578	8.497	0.447	0.083	0.833
IL12B	7.160	6.996	6.642	0.395	0.004	0.202
MMP10	7.850	7.615	7.034	0.563	0.004	0.201
IL10	4.574	4.077	4.006	0.112	0.013	0.901
TNF	3.574	3.248	3.247	0.366	0.083	>0.999
CCL23	9.783	9.767	9.896	>0.999	0.407	0.789
CD5	6.214	5.664	5.479	0.090	0.004	0.612
CCL3	5.443	5.253	4.798	0.617	0.006	0.419
FLT3LG	9.140	8.879	8.907	0.213	0.042	0.964
CXCL6	10.300	9.498	9.033	0.338	0.014	0.615
CXCL10	9.522	9.496	9.460	>0.999	0.555	0.964
EIF4EBP1	9.352	8.260	8.246	0.250	0.048	>0.999
SIRT2	7.331	5.565	5.452	0.187	0.026	0.964
CCL28	2.595	2.191	1.926	0.187	0.004	0.425
S100A12	3.697	3.666	3.034	>0.999	0.054	0.414
CD40	11.890	11.530	11.430	0.356	0.056	0.901
IFN-gamma	6.590	6.741	6.670	0.816	0.602	0.964
FGF19	8.408	7.809	8.019	0.213	0.199	0.789
CCL8	9.429	8.984	8.916	0.392	0.089	0.964
CASP8	4.083	3.042	2.305	0.315	0.012	0.453
CCL25	5.720	5.636	5.698	0.857	0.656	0.921
CX3CL1	6.222	6.323	6.379	0.617	0.186	0.901
TNFRSF9	8.190	7.896	8.047	0.099	0.214	0.493
NTF3	3.032	3.014	3.102	>0.999	0.402	0.789
TNFSF12	10.410	10.120	10.040	0.187	0.006	0.789
CCL20	7.574	7.241	7.041	0.315	0.013	0.603
SULT1A1	4.791	3.946	3.508	0.366	0.039	0.730
STAMBP	7.346	6.142	5.769	0.250	0.020	0.798
ADA	6.272	6.113	5.850	0.685	0.083	0.464
TNFB	6.037	5.591	5.613	0.061	0.006	0.964
CSF1	10.020	10.110	10.140	0.464	0.083	0.901
DNER	9.617	9.414	9.360	0.061	0.006	0.789
CD8A	10.620	10.370	10.450	0.410	0.307	0.901

Supplementary Table S3. Random forest analysis identified the proteins' contribution to the separation of the three groups.

Random forest analysis resulted in a predictive accuracy of 90.6%. The higher the importance, the more the protein contributes to the separation of active sJIA, inactive sJIA and healthy control.

Protein name	Importance
**IL6**	**0.2338**
**KITLG**	**0.1962**
**IL18**	**0.1038**
**TNFB**	**0.0320**
**CXCL1**	**0.0308**
**CCL19**	**0.0294**
**CCL23**	**0.0293**
**S100A12**	**0.0260**
**MMP1**	**0.0228**
**PLAU**	**0.0204**
**CCL2**	**0.0194**
**CXCL5**	**0.0188**
**CST5**	**0.0152**
**OSM**	**0.0143**
**CXCL11**	**0.0126**
**FGF23**	**0.0101**
CCL13	0.0099
SULT1A1	0.0093
IL18R1	0.0089
TNFRSF9	0.0080
FLT3LG	0.0075
CCL11	0.0075
CDCP1	0.0073
TNFSF14	0.0066
TGFA	0.0065
OPG	0.0063
CXCL9	0.0061
CSF1	0.0057
IL10	0.0054
TNFSF10	0.0054
NTF3	0.0042
TNFSF12	0.0042
IL12B	0.0040
IL7	0.0038
CD8A	0.0037
FGF19	0.0036
ADA	0.0035
CD40	0.0034
DNER	0.0034
TNFSF11	0.0032
MMP10	0.0030
CCL20	0.0028
CASP8	0.0028
CCL25	0.0027
CD274	0.0027
IL8	0.0024
IL15RA	0.0022
CCL4	0.0022
EIF4EBP1	0.0020
SIRT2	0.0020
IL10RB	0.0018
VEGFA	0.0018
FGF21	0.0018
CCL28	0.0017
AXIN1	0.0016
CD5	0.0016
CCL8	0.0016
CXCL6	0.0014
CX3CL1	0.0013
TGFB1	0.0012
CD6	0.0011
CD244	0.0010
IL10RA	0.0009
HGF	0.0009
IL17A	0.0009
CXCL10	0.0007
LIFR	0.0006
CCL3	0.0006

Supplementary Table S4. Detailed fold change and p-values between the cross-sectional comparisons.

Two-way ANOVA with correction of multiple comparison by controlling the False Discovery Rate (FDR) of 5% via two-stage step-up method of Benjamini, Krieger and Yekutieli. Fold change was calculated as dividing the average NPX values in active or inactive sJIA by the average NPX values in age- and gender-matched healthy controls. The significant factors are highlighted in red.

Protein name	Fold changes (active sJIA/healthy control)	Adjust p value (active sJIA/healthy control)	Protein name	Fold changes (inactive sJIA/healthy control)	Adjust p value (inactive sJIA/healthy control)
**IL6**	**2.1338**	**<0.0001**	**CXCL5**	**0.8760**	**<0.0001**
**OSM**	**1.5089**	**<0.0001**	**SULT1A1**	**0.7012**	**0.0001**
**KITLG**	**0.8346**	**<0.0001**	**IL18**	**1.1213**	**0.0012**
**IL18**	**1.2604**	**<0.0001**	**CASP8**	**0.6842**	**0.0014**
**MMP1**	**1.2612**	**<0.0001**	**CXCL1**	**0.8973**	**0.0018**
**S100A12**	**1.6699**	**<0.0001**	**SIRT2**	**0.8671**	**0.0304**
**CXCL11**	**1.1536**	**0.0008**	IL7	0.8043	0.0553
**CD6**	**0.8509**	**0.0160**	CCL13	0.9453	0.0553
**TNFSF11**	**0.8435**	**0.0200**	STAMBP	0.8908	0.0736
**EIF4EBP1**	**1.1071**	**0.0261**	AXIN1	0.8693	0.1825
FGF21	1.3101	0.0583	MMP1	0.9379	0.2218
IL10	1.1933	0.0583	CXCL6	0.9426	0.2218
CD5	0.8619	0.0583	CCL8	0.9393	0.2218
TNFRSF9	0.9007	0.0583	IL8	0.9033	0.2600
SULT1A1	0.8102	0.0682	TNFSF14	0.8904	0.3364
IL12B	0.8889	0.0719	CCL23	0.9581	0.4917
TNFB	0.8746	0.0922	CD8A	1.0396	0.4917
VEGFA	1.0665	0.1052	CD6	0.9460	0.6594
HGF	1.0797	0.1074	EIF4EBP1	1.0422	0.6594
TNFSF14	1.1508	0.1348	CCL25	1.0615	0.6594
CCL2	1.0548	0.1792	CST5	1.0573	0.6620
CCL23	1.0602	0.1824	KITLG	0.9681	0.6620
TNFSF12	0.9437	0.1958	CCL11	1.0504	0.6620
SIRT2	1.0928	0.1978	CCL19	0.9677	0.6620
CDCP1	1.1897	0.2095	CD40	0.9739	0.6620
IL18R1	1.0663	0.2095	FGF19	1.0367	0.7014
MMP10	0.9320	0.2742	CXCL9	1.0396	0.7202
CCL19	0.9509	0.2820	OSM	1.0676	0.7202
CCL3	1.0954	0.2820	CCL4	0.9566	0.7202
FGF19	0.9412	0.2820	IL10	1.0682	0.7202
PLAU	0.9542	0.2833	TGFB	0.9674	0.7564
CXCL6	0.9518	0.2944	TRAIL	1.0262	0.8653
CXCL10	1.0488	0.2944	VEGFA	0.9797	0.8779
FIT3LG	0.9495	0.2952	CD5	0.9647	0.8779
CASP8	0.8698	0.3355	OPG	0.9819	0.9147
CCL4	1.0652	0.3706	MMP10	1.0252	0.9147
CD274	1.0565	0.3988	ADA	0.9699	0.9147
TGFA	1.1013	0.3991	CCL2	1.0149	0.9338
IL17A	1.1922	0.4385	CDCP1	0.9534	0.9413
DNER	0.9651	0.4608	CD244	0.9858	0.9413
FGF23	1.1080	0.4998	PLAU	1.0069	0.9413
CD8A	1.0287	0.4998	IL17A	1.0409	0.9413
NT3	0.9011	0.5053	CXCL11	1.0126	0.9413
CXCL1	1.0282	0.5339	TGFA	1.0357	0.9413
CSF1	1.0269	0.5480	FGF23	1.0470	0.9413
TGFB	1.0333	0.5636	IL10RA	1.0916	0.9413
CXCL5	0.9769	0.5636	CD274	0.9817	0.9413
CCL8	1.0256	0.6001	TNFSF11	1.0217	0.9413
IL7	0.9428	0.6051	HGF	0.9915	0.9413
IL10RA	1.1769	0.6051	IL12B	0.9769	0.9413
CCL20	1.0310	0.6051	CCL3	0.9794	0.9413
TRAIL	0.9765	0.6440	FIT3LG	1.0070	0.9413
CD244	0.9749	0.6443	CXCL10	0.9911	0.9413
CX3CL1	0.9705	0.6443	S100A12	1.0148	0.9413
CCL11	1.0280	0.6564	CX3CL1	1.0115	0.9413
STAMBP	1.0266	0.6564	TNFRSF9	0.9892	0.9413
IL8	1.0288	0.6856	NT3	1.0178	0.9413
CCL25	0.9772	0.7337	TNFSF12	0.9905	0.9413
AXIN1	0.9740	0.7470	CCL20	0.9840	0.9413
CXCL9	1.0148	0.7804	CSF1	0.9938	0.9413
IL10RB	0.9862	0.7804	FGF21	1.0110	0.9480
CD40	1.0069	0.7804	IL15RA	0.9745	0.9480
ADA	0.9857	0.7804	IL18R1	0.9953	0.9480
CST5	0.9902	0.8309	DNER	0.9970	0.9480
LIFR	1.0125	0.8309	IL6	1.0032	0.9524
IL15RA	0.9625	0.8309	LIFR	0.9995	0.9524
CCL13	1.0023	0.8410	IL10RB	1.0013	0.9524
CCL28	0.9847	0.8410	CCL28	1.0074	0.9524
OPG	0.9994	0.8861	TNFB	1.0004	0.9524

Supplementary Table S5. Detailed fold change and p-Values between active and inactive sJIA paired analysis.

Two-way ANOVA with correction of multiple comparison by controlling the False Discovery Rate (FDR) of 5% via two-stage step-up method of Benjamini, Krieger and Yekutieli. Fold change was calculated as dividing the average NPX values in active sJIA by the average NPX values in the paired inactive sJIA. The significant factors are highlighted in red.

Protein	Fold changes (active sJIA/ inactive sJIA)	Adjust p value (active sJIA/ inactive sJIA)
**IL6**	**1.8729**	**<0.0001**
**MMP1**	**1.2739**	**<0.0001**
**S100A12**	**1.8258**	**<0.0001**
**OSM**	**1.4013**	**0.0014**
**CXCL11**	**1.1956**	**0.0017**
**SIRT2**	**1.3056**	**0.0017**
**CXCL5**	**1.1414**	**0.0077**
**CXCL1**	**1.1524**	**0.0105**
**KITLG**	**0.8673**	**0.0163**
**TNFSF11**	**0.8135**	**0.0267**
**EIF4EBP1**	**1.1370**	**0.0281**
TNFSF14	1.2850	0.0517
STAMBP	1.1913	0.0519
CCL23	1.1002	0.1140
IL7	1.3254	0.1254
VEGFA	1.0864	0.1390
HGF	1.0898	0.2114
CXCL6	1.0880	0.2114
CCL8	1.0880	0.2114
CASP8	1.3848	0.2114
TNFRSF9	0.9072	0.2114
SULT1A1	1.3210	0.2114
IL8	1.1515	0.2232
CD6	0.8958	0.2694
IL12B	0.9018	0.2694
IL18	1.0640	0.3067
IL10	1.1379	0.3520
AXIN1	1.1468	0.4025
CCL13	1.0426	0.4025
CD5	0.8990	0.4025
CCL20	1.0793	0.4025
CDCP1	1.1817	0.4481
FGF19	0.9399	0.4481
IL17A	1.2742	0.4996
TGFB	1.0572	0.5512
CCL4	1.0711	0.5512
IL18R1	1.0533	0.5512
CCL3	1.0848	0.5512
CXCL10	1.0457	0.5512
CCL25	0.9313	0.5726
FIT3LG	0.9579	0.6116
CD274	1.0533	0.6142
MMP10	0.9495	0.6142
TNFB	0.9380	0.6142
OPG	1.0357	0.6227
ADA	1.0605	0.6227
CST5	0.9427	0.6345
CCL2	1.0287	0.6510
TRAIL	0.9656	0.6922
CX3CL1	0.9578	0.7032
TNFSF12	0.9728	0.7032
CSF1	1.0274	0.7032
PLAU	0.9762	0.7415
TGFA	1.0499	0.8102
CD40	1.0158	0.8102
DNER	0.9806	0.8102
CD8A	0.9844	0.8116
NT3	0.9498	0.8534
CXCL9	0.9800	0.8571
CD244	0.9844	0.8585
CCL11	0.9894	0.8585
FGF23	1.0222	0.8585
IL10RA	1.0822	0.8585
CCL19	1.0108	0.8585
IL15RA	0.9461	0.8585
IL10RB	0.9849	0.8585
CCL28	1.0286	0.8585
FGF21	0.9872	0.8781
LIFR	0.9987	0.9015

Supplementary Table S6. Top cellular functions results from comparison between active sJIA and healthy controls.

Diseases or Functions Annotation	p-value	Predicted Activation State	Activation z-score	Molecules	# Molecules
Growth of connective tissue	2.68E-07		-1.184	CD6,IL18,IL6,KITLG,OSM	5
Hematopoiesis of mononuclear leukocytes	4.26E-08		-0.653	CD6,CXCL11,IL6,KITLG,TNFSF11	5
Cell survival	8.37E-04		-0.440	CXCL11,IL6,KITLG,OSM,TNFSF11	5
Adhesion of immune cells	3.44E-08		-0.058	CD6,IL18,IL6,KITLG,TNFSF11	5
Apoptosis of tumor cell lines	9.00E-05		0.158	EIF4EBP1,IL18,IL6,KITLG,OSM,TNFSF11	6
Activation of leukocytes	3.37E-07		0.225	CD6,IL18,IL6,S100A12,TNFSF11	5
Binding of leukocytes	6.16E-10		0.276	CD6,CXCL11,IL18,IL6,KITLG,TNFSF11	6
Cell movement of leukocytes	1.60E-08		0.715	CXCL11,IL18,IL6,KITLG,S100A12,TNFSF11	6
Leukocyte migration	1.73E-09		0.766	CXCL11,IL18,IL6,KITLG,MMP1,S100A12,TNFSF11	7
Cellular homeostasis	7.76E-06		0.794	CD6,EIF4EBP1,IL18,IL6,KITLG,MMP1	6
Activation of cells	1.88E-09		0.911	CD6,IL18,IL6,MMP1,OSM,S100A12,TNFSF11	7
Migration of tumor cell lines	5.89E-07		1.076	CXCL11,EIF4EBP1,IL18,IL6,KITLG,MMP1,OSM	7
Invasion of cells	2.35E-04		1.194	CXCL11,EIF4EBP1,IL18,IL6,MMP1	5
Migration of cells	1.68E-08		1.201	CXCL11,EIF4EBP1,IL18,IL6,KITLG,MMP1,OSM,S100A12,TNFSF11	9
Expression of RNA	2.68E-03		1.213	EIF4EBP1,IL18,IL6,OSM,TNFSF11	5
Inflammatory response	4.19E-10		1.626	CXCL11,IL18,IL6,KITLG,OSM,S100A12,TNFSF11	7

Supplementary Table S7. Top cellular functions results from comparison between inactive sJIA and healthy controls.

Diseases or Functions Annotation	p-value	Predicted Activation State	Activation z-score	Molecules	# Molecules
Cell movement of tumor cell lines	4.69E-04		-1.118	CASP8,CXCL1,CXCL5,IL18	4
Migration of cells	1.66E-03		-0.856	CASP8,CXCL1,CXCL5,IL18	4
Cellular homeostasis	4.35E-06		-0.037	CASP8,CXCL1,CXCL5,IL18,SIRT2	5
Necrosis	4.71E-03		-0.025	CASP8,CXCL1,IL18,SIRT2	4

Supplementary Table S8. Top cellular functions results from comparison between active sJIA and inactive sJIA.

Diseases or Functions Annotation	p-value	Predicted Activation State	Activation z-score	Molecules	# Molecules
Inflammatory response	1.27E-11	Increased	2.140	CXCL1,CXCL11,CXCL5,IL6,KITLG,OSM,S100A12,TNFSF11	8
Invasion of cells	1.38E-03		1.934	CXCL1,CXCL5,IL6,MMP1	4
Chemotaxis of leukocytes	1.63E-11		1.648	CXCL1,CXCL11,CXCL5,IL6,KITLG,S100A12,TNFSF11	7
Migration of cells	6.33E-11		1.442	CXCL1,CXCL11,CXCL5,EIF4EBP1,IL6,KITLG,MMP1,OSM,S100A12,TNFSF11	10
Leukocyte migration	5.53E-11		1.396	CXCL1,CXCL11,CXCL5,IL6,KITLG,MMP1,S100A12,TNFSF11	8
Cellular homeostasis	1.61E-07		0.782	CXCL1,CXCL5,EIF4EBP1,IL6,KITLG,MMP1,SIRT2	7
Cell cycle progression	1.22E-05		0.772	EIF4EBP1,IL6,KITLG,OSM,SIRT2	5
Activation of cells	1.27E-04		0.720	IL6,OSM,S100A12,TNFSF11	4
Chemotaxis of phagocytes	3.61E-10		0.556	CXCL1,CXCL11,CXCL5,KITLG,S100A12,TNFSF11	6
Cell movement of myeloid cells	3.09E-09		0.470	CXCL1,CXCL5,IL6,KITLG,S100A12,TNFSF11	6
Cell viability	2.76E-04		0.195	IL6,KITLG,OSM,TNFSF11	4
Activation of DNA endogenous promoter	1.94E-03		0.068	IL6,OSM,SIRT2,TNFSF11	4
Apoptosis	1.03E-04		-0.059	CXCL1,EIF4EBP1,IL6,KITLG,OSM,TNFSF11	6
Cell movement of phagocytes	3.52E-11		-0.078	CXCL1,CXCL11,CXCL5,IL6,KITLG,S100A12,TNFSF11	7
Expression of RNA	2.87E-03		-0.090	EIF4EBP1,IL6,OSM,SIRT2,TNFSF11	5
Hematopoiesis of mononuclear leukocytes	6.67E-08		-0.152	CXCL11,CXCL5,IL6,KITLG,TNFSF11	5
Cell survival	1.90E-05		-0.329	CXCL11,IL6,KITLG,OSM,TNFSF11	5
Binding of leukocytes	5.46E-06		-0.437	CXCL11,IL6,KITLG,TNFSF11	4
Transmigration of leukocytes	8.53E-08		-0.492	CXCL11,CXCL5,IL6,KITLG	4
Necrosis	4.68E-04		-0.988	CXCL1,IL6,KITLG,OSM,TNFSF11	5
Cell death of immune cells	1.34E-05		-1.185	CXCL1,IL6,KITLG,TNFSF11	4
Development of connective tissue cells	6.04E-08		-1.403	CXCL11,IL6,KITLG,TNFSF11	4
Monocytopoiesis	1.33E-07		-1.448	CXCL11,IL6,KITLG,TNFSF11	4

Supplementary Table S9. Top canonical pathways results from comparison between active sJIA and healthy controls.
Z-score NaN means no z-score could be calculated based on the input factors.

Ingenuity Canonical Pathways	-log(p-value)	Ratio	z-score	Molecules
Airway Pathology in Chronic Obstructive Pulmonary Disease	8.990	0.044	NaN	IL18,IL6,MMP1,OSM,TNFSF11
Role of Macrophages, Fibroblasts and Endothelial Cells in Rheumatoid Arthritis	6.770	0.016	1.000	IL18,IL6,MMP1,OSM,TNFSF11
Role of Pattern Recognition Receptors in Recognition of Bacteria and Viruses	6.340	0.027	NaN	IL18,IL6,OSM,TNFSF11
HMGB1 Signaling	6.190	0.025	0.556	IL18,IL6,OSM,TNFSF11
Erythropoietin Signaling Pathway	6.090	0.024	-1.000	IL18,IL6,OSM,TNFSF11
IL-17 Signaling	5.980	0.022	1.000	IL18,IL6,OSM,TNFSF11
Hepatic Cholestasis	5.950	0.022	NaN	IL18,IL6,OSM,TNFSF11
Cardiac Hypertrophy Signaling (Enhanced)	5.650	0.009	1.000	EIF4EBP1,IL18,IL6,OSM,TNFSF11
Role of Osteoblasts, Osteoclasts and Chondrocytes in Rheumatoid Arthritis	5.630	0.018	NaN	IL18,IL6,MMP1,TNFSF11
Systemic Lupus Erythematosus In B Cell Signaling Pathway	5.290	0.015	1.000	IL18,IL6,OSM,TNFSF11

Supplementary Table S10. Top canonical pathways results from comparison between inactive sJIA and healthy controls.
Z-score NaN means no z-score could be calculated based on the input factors.

Ingenuity Canonical Pathways	-log(p-value)	Ratio	z-score	Molecules
Role of IL-17A in Psoriasis	5.210	0.143	NaN	CXCL1,CXCL5
Granulocyte Adhesion and Diapedesis	4.960	0.017	NaN	CXCL1,CXCL5,IL18
IL-17 Signaling	4.920	0.017	NaN	CXCL1,CXCL5,IL18
Inflammasome pathway	4.890	0.100	0.342	CASP8,IL18
Agranulocyte Adhesion and Diapedesis	4.800	0.015	NaN	CXCL1,CXCL5,IL18
Role of IL-17F in Allergic Inflammatory Airway Diseases	4.190	0.046	NaN	CXCL1,CXCL5
Role of IL-17A in Arthritis	3.970	0.035	NaN	CXCL1,CXCL5
IL-17A Signaling in Airway Cells	3.820	0.030	NaN	CXCL1,CXCL5
Airway Pathology in Chronic Obstructive Pulmonary Disease	3.370	0.018	NaN	CXCL1,IL18
Role of PKR in Interferon Induction and Antiviral Response	3.240	0.015	NaN	CASP8,IL18

Supplementary Table S11. Top canonical pathways results from comparison between active sJIA and inactive sJIA.
Z-score NaN means no z-score could be calculated based on the input factors.

Ingenuity Canonical Pathways	-log(p-value)	Ratio	z-score	Molecules
IL-17 Signaling	7.720	0.028	1.342	CXCL1,CXCL5,IL6,OSM,TNFSF11
Airway Pathology in Chronic Obstructive Pulmonary Disease	8.730	0.044	NaN	CXCL1,IL6,MMP1,OSM,TNFSF11
IL-17A Signaling in Fibroblasts	6.050	0.079	NaN	CXCL5,IL6,MMP1
Role of IL-17F in Allergic Inflammatory Airway Diseases	5.850	0.068	NaN	CXCL1,CXCL5,IL6
Granulocyte Adhesion and Diapedesis	5.840	0.023	0.342	CXCL1,CXCL11,CXCL5,MMP1
Agranulocyte Adhesion and Diapedesis	5.630	0.020	NaN	CXCL1,CXCL11,CXCL5,MMP1
Role of IL-17A in Arthritis	5.510	0.053	NaN	CXCL1,CXCL5,MMP1
IL-17A Signaling in Airway Cells	5.300	0.045	NaN	CXCL1,CXCL5,IL6
Role of Macrophages, Fibroblasts and Endothelial Cells in Rheumatoid Arthritis	4.830	0.013	NaN	IL6,MMP1,OSM,TNFSF11
Role of IL-17A in Psoriasis	4.640	0.143	NaN	CXCL1,CXCL5

Supplementary Figures

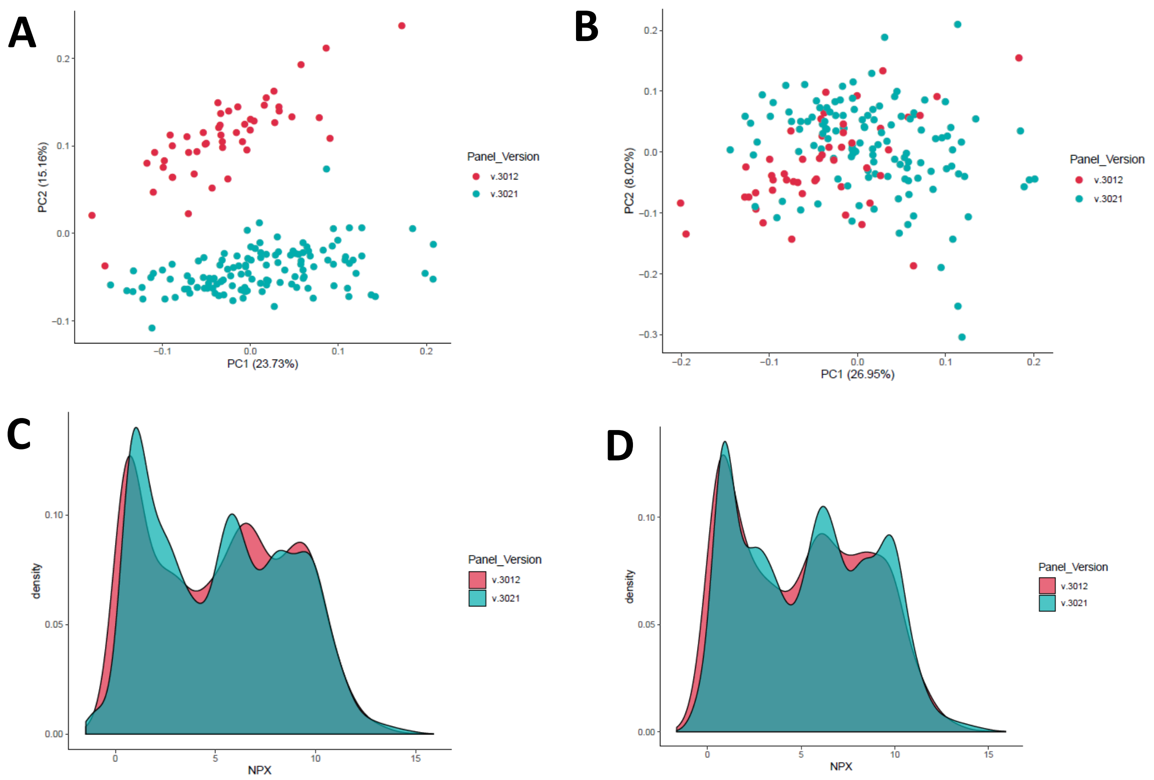

Supplementary Fig. S1. Normalization of two plasma datasets from different inflammation panel versions. PCA plot of plasma samples before (A) and after (B) normalization colored by panel version. The percentage in parenthesis on the axis labels represent the percent of variance explained by that principal component. Density plot of the NPX values from the two plasma datasets before (C) and after (D) normalization colored by panel version. The normalization removed some of the grouping in the PCA plots, while the changes in the distribution and density plots were not as obvious. The normalization was performed and the Figures were provided as a report by Olink statistical service.

A

	4 years old	8 years old	12 years old
**Number of sample**	20	20	20
**Gender (Female %)**	60%	50%	75%

B

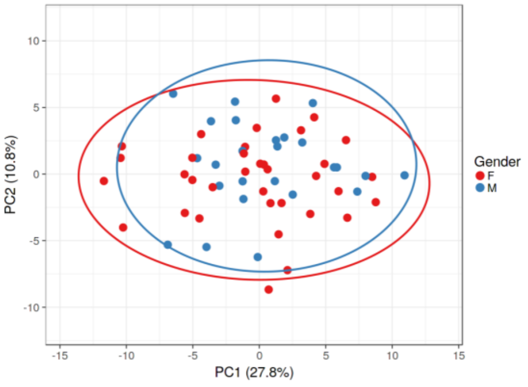

C

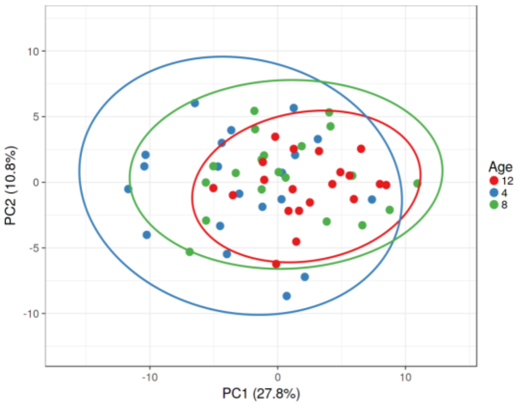

Supplementary Fig. S2. Age is a major confounding factor and gender also matters. (A) Number and gender of different-aged healthy control. (B) PCA analysis of 60 healthy control based on gender. (C) PCA analysis of 60 healthy control based on age. The confidence level of the ellipses is 0.95.

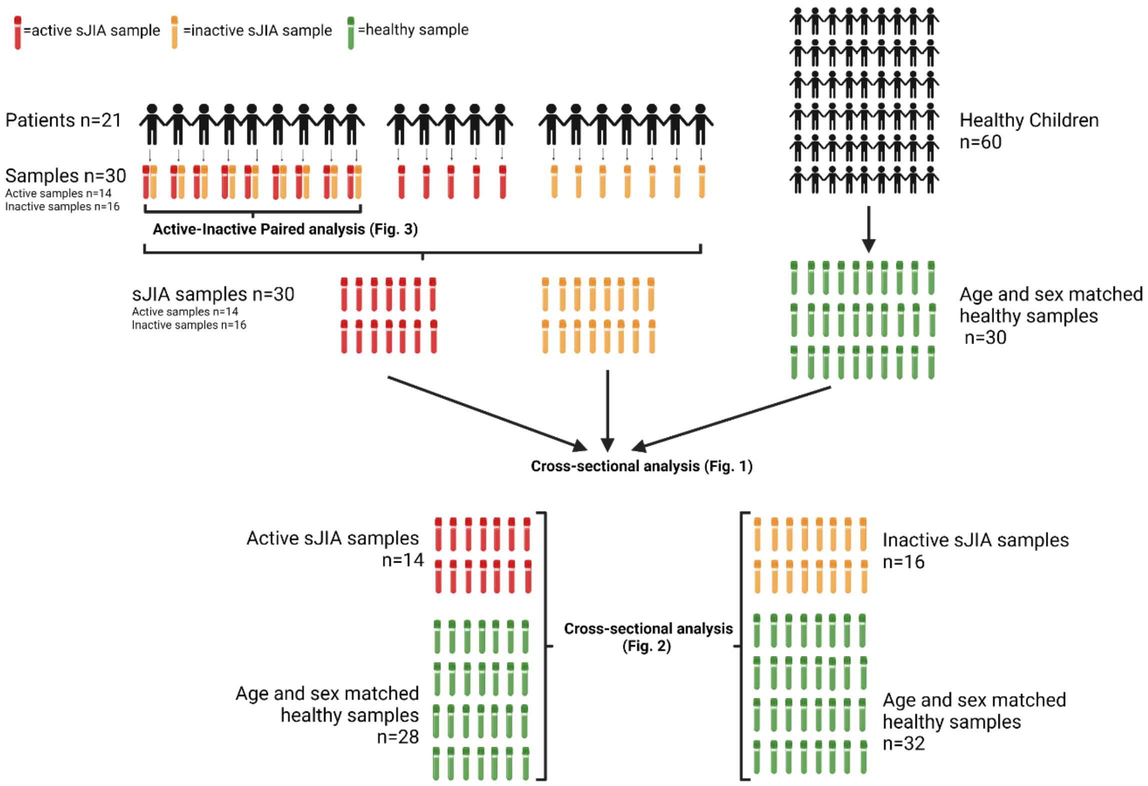

Supplementary Fig. S3. Illustration of analysis settings in this study. In the cross-sectional analysis, ordinary two-way ANOVA was performed on active sJIA (n=14), inactive sJIA (n=16) and healthy controls (n=30). Multiple t-test was performed on active sJIA (n=14) versus healthy controls (n=28) and on inactive sJIA (n=16) versus healthy controls (n=32), separately. In each cross-sectional analysis, the healthy control group was age- and gender-matched to the patient group. There are in total 60 healthy samples. In each cross-sectional analysis, we always try our best to select samples which are best sex and gender match to the patient sample. Therefore, in the three cross-sectional analysis, the healthy samples number are 30, 28 and 32 separately. In the paired analysis, two-way repeat-measurement ANOVA was performed on paired active sJIA (n=9) and inactive sJIA (n=9) samples from 9 patients. All the statistical analyses were corrected for multiple comparison by controlling the False Discovery Rate (FDR) via two-stage step-up method of Benjamini, Krieger and Yekutieli. Adjusted p-values less than 0.05 were regarded as significant.

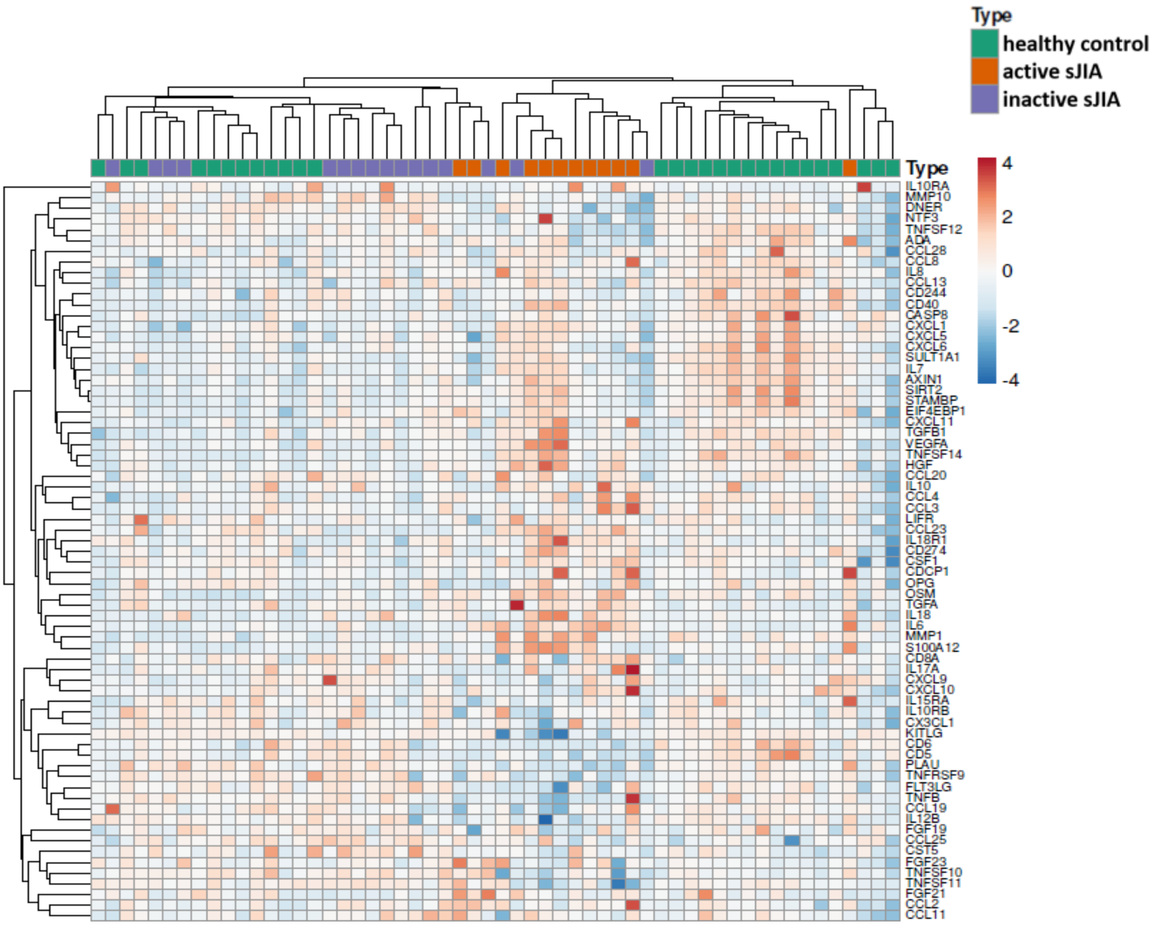

Supplementary Fig. S4. Distribution of the different subgroups based on 69 detected inflammation-associated proteins. Hierarchical clustering analysis showing the grouping among active sJIA, inactive sJIA and controls. Unit variance scaling was applied to rows; both rows and columns were clustered using correlation distance and average linkage.

References

1. Assarsson E, Lundberg M, Holmquist G, Björkesten J, Thorsen SB, Ekman D, et al. Homogenous 96-Plex Pea Immunoassay Exhibiting High Sensitivity, Specificity, and Excellent Scalability. *PLoS One* 2014;9:e95192.
